# Therapeutic cancer vaccines: advancements, challenges and prospects

**DOI:** 10.1038/s41392-023-01674-3

**Published:** 2023-12-13

**Authors:** Ting Fan, Mingna Zhang, Jingxian Yang, Zhounan Zhu, Wanlu Cao, Chunyan Dong

**Affiliations:** 1 Department of Oncology, East Hospital Affiliated to Tongji University, Tongji University School of Medicine, Shanghai, China; 2 Postgraduate Training Base, Shanghai East Hospital, Jinzhou Medical University, Shanghai, 200120 China

**Keywords:** Drug development, Drug development

## Abstract

With the development and regulatory approval of immune checkpoint inhibitors and adoptive cell therapies, cancer immunotherapy has undergone a profound transformation over the past decades. Recently, therapeutic cancer vaccines have shown promise by eliciting de novo T cell responses targeting tumor antigens, including tumor-associated antigens and tumor-specific antigens. The objective was to amplify and diversify the intrinsic repertoire of tumor-specific T cells. However, the complete realization of these capabilities remains an ongoing pursuit. Therefore, we provide an overview of the current landscape of cancer vaccines in this review. The range of antigen selection, antigen delivery systems development the strategic nuances underlying effective antigen presentation have pioneered cancer vaccine design. Furthermore, this review addresses the current status of clinical trials and discusses their strategies, focusing on tumor-specific immunogenicity and anti-tumor efficacy assessment. However, current clinical attempts toward developing cancer vaccines have not yielded breakthrough clinical outcomes due to significant challenges, including tumor immune microenvironment suppression, optimal candidate identification, immune response evaluation, and vaccine manufacturing acceleration. Therefore, the field is poised to overcome hurdles and improve patient outcomes in the future by acknowledging these clinical complexities and persistently striving to surmount inherent constraints.

## Introduction

Overcoming malignant tumors, which are the primary cause of mortality, is crucial to increase global life expectancy. A staggering estimate of 19.3 million novel cancer cases and an unfortunate toll of approximately 10 million cancer-related mortalities were witnessed in 2020, highlighting the urgency of this challenge.^1^ Conventional cancer therapies, including surgical interventions, radiotherapy, and chemotherapy, are substantially toxic and exhibit restricted applicability, thereby underscoring the urgency for developing more efficacious cancer treatment modalities.^2^ Current relevant studies suggest that cancer progressing is highly associated with “cancer immunoediting”. This dynamic interplay indicates that the immune system is able to eradicate nascent cancer cells by recognizing mutated oncogenic genes or foster an immunosuppressive microenvironment conducive to tumor proliferation.^3^ Therefore, the fate of cancer cells is determined by a delicate balance within the immune system.

**Fig. 1:**
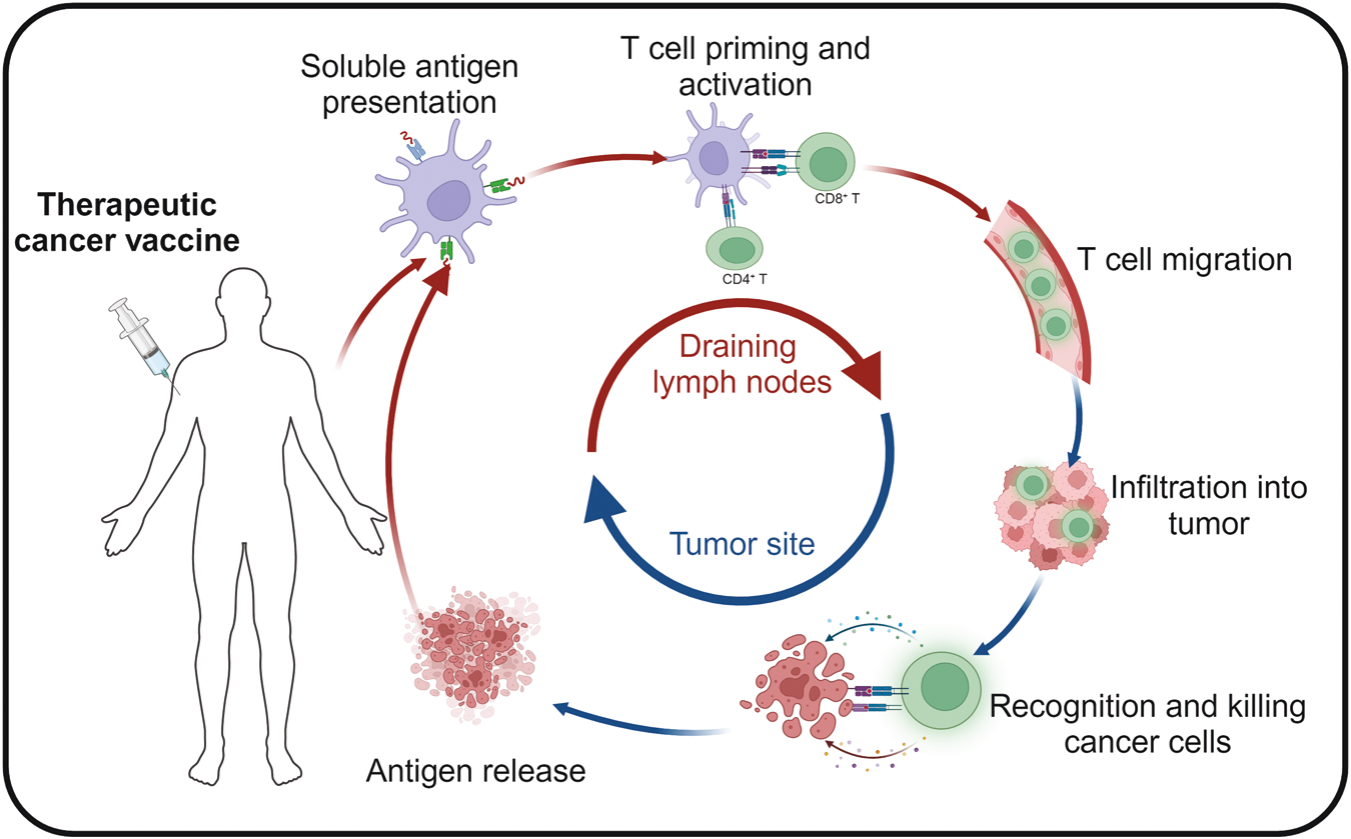
The mechanism of cancer vaccine in vivo. After the tumor antigens migrate into the body in different forms, they are phagocytosed, intracellularly expressed, and efficiently processed by specialized antigen-presenting cells (APCs). The major histocompatibility complex (MHC) of dendritic cells presents antigens to their surface, and the MHC complexes activate antigen-specific T-cells by binding to T-cell receptors (TCR) on the surface of T-cells, therefore safely, persistently, and specifically destroying tumor cells and inhibiting tumor growth

Recently, immunotherapy has taken center stage in the fight against cancer. Several novel immunotherapies, including immune checkpoint inhibitors (ICIs), oncolytic viruses, and chimeric antigen receptor-T cell therapies, have been licensed for clinical use.^4–6^ ICIs have become the most promising immunotherapy type since the Food and Drug Administration (FDA)’s first approval of cytotoxic T-lymphocyte-associated antigen 4 antibody in 2011. According to Haslam’s data, although 43% of patients with cancer meet the indications for ICIs use, only 12% benefit from the treatment.^7^ Therefore, exploring novel immunotherapeutic approaches, including therapeutic cancer vaccines, to address this issue has gained increasing interest. Vaccines were originally conceived for the primary purpose of averting infectious diseases. Nevertheless, their capacity to enhance antigen-specific immune reactions has gained recognition as a promising therapeutic instrument for combating cancer.^8^ Although vaccines can incorporate predefined or unknown antigens, this study focused on predefined cancer vaccines, including shared antigens or personalized neoantigens. After immunization, the cancer antigen is absorbed by the antigen presentation cells (APCs), where tumor-related antigens are processed into major histocompatibility complex (MHC) I/II complexes. Subsequently, the activated APCs undergo migration into the draining lymph nodes, where MHC I/II complexes bind to T cells, causing their priming and activation. The activated T cells travel towards the tumor site, infiltrating the tumor tissue under favorable co-stimulatory conditions and guided by chemokine gradients. Once within the tumor microenvironment, these activated T cells can control tumor growth through direct tumor cell destruction and cytokine-mediated processes (Fig. 1).^9^

**Fig. 2:**
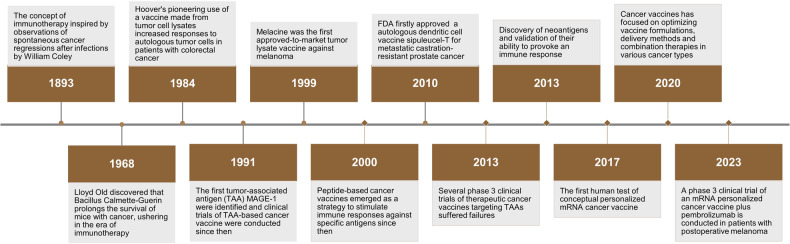
The history and the key time points of therapeutic cancer vaccines

Therapeutic cancer vaccine development has experienced numerous advancements and drawbacks over the past century, beginning with William Coley’s early efforts to employ inactivated bacteria to kill tumor cells. The history and the key time point of therapeutic cancer vaccines could be found in Fig. 2. Despite the progress in this field, further exploration is required to improve the accessibility and effectiveness of cancer-active immunotherapies. This is particularly important considering these treatments’ high cost and limited availability, restricting the number of individuals who can benefit from them. The ultimate objective of cancer vaccines is to prime antigen-specific T cells, which are indispensable for an effective immune response against cancer.^10^ Current clinical studies on cancer vaccines are challenging and have not yielded remarkable clinical outcomes.^11^ However, innovative strategies and technological advancements present promising prospects to overcome these challenges and broaden the opportunities for clinical applications.

In this review, we provide a comprehensive overview of the current cancer vaccines. First, we describe strategies for antigen repertoire selection and delivery platforms to improve cancer vaccine design. We subsequently review ongoing clinical trials and explore future advancements in this field by exploring key areas, such as neoantigens identification and selection, innovative vaccine platform development, and strategies for enhancing the antigen-specific T cell response. Additionally, we summarize the most recent clinical advancements in cancer vaccine research and provide a future perspective on the challenges and opportunities in this promising novel immunotherapy.

## Optimal tumor antigen selection in cancer vaccine

Ideally, candidate tumor antigen for cancer vaccines have to exhibit elevated expression in tumor tissue. Tumor antigens are categorized into shared and personalized antigens based on their expression frequency.^12^ Shared antigens are “public” antigens containing hotspot mutations by a relatively common human leukocyte antigen (HLA) allele in patients.^13^ They target tumor-associated antigens (TAA) and tumor-specific antigens (TSA). TAA is an autoantigen expressed in normal tissues and overexpressed in various cancers, including cancer-testis antigens, tissue-differentiation neoantigens, and overexpression antigens.^14–16^ Contrarily, TSA is directly produced from numerous non-synonymous somatic mutations that can increase MHC presentation to antigen epitopes or alter their T cell receptor (TCR) recognition. Melanoma-associated antigen (MAGE) is normally expressed and overexpressed in the testis and melanoma, respectively, whereas human papillomavirus (HPV)-associated cervical and oropharyngeal cancers have high expression of the E6 and E7 proteins of high-risk HPV.^17,18^ Therefore, the shared TAAs in patients with cancer make it a promising off-the-shelf immunotherapy option. Personalized cancer vaccines have recently gained the spotlight due to modern high-throughput gene sequencing technology development and a deeper understanding of neoantigens production. These TSAs are generally not germ line-encoded and rarely cause immune tolerance, rendering them ideal as tumor immunotherapy targets.^19^ Immunogenic neoantigens selection must be performed using complementarity algorithms considering several factors. However, vaccine production time, vaccine design costs, and subsequent personalized neoantigen pool generation pose significant challenges for the large-scale implementation of this technology.

### Shared antigen cancer vaccine

Shared antigen vaccines target antigens commonly expressed across multiple cancer types compared to personalized cancer vaccines targeting specific mutations in an individual’s tumor, making them off-the-shelf and low-cost immunotherapies.

#### Tumor-associated antigen

Shared antigen vaccines can be developed using these two approaches.^11^ One common strategy involves employing TAAs, which are proteins highly expressed by cancer cells. An illustrative instance is the first approved autologous dendritic cell vaccine, sipuleucel-T, which prolonged survival of 2–4 months in patients with metastatic resistant prostate cancer. This therapeutic intervention targets prostate acid phosphatase, a TAA that exhibits high expression levels in prostate cancer cells.^20,21^ Additionally, a messenger RNA (mRNA) vaccine comprising four TAAs has demonstrated the capacity to elicit robust and durable immune responses directed against these antigens, with or without ICI, in patients with unresectable melanoma.^22^ A lipid nanoparticle (LNP)-based cancer vaccine encoding the four most frequent Kirsten rat sarcoma virus (KRAS) mutation antigens (G12D, G12V, G13D, and G12C) is able to elicit both cytotoxic and memory T cell phenotypes targeting KRAS-mutated tumor cells. Currently, a phase 1 clinical trial is carried out in patients with advanced KRAS-mutated cancers (NCT03948763).^23^ Wilms’ tumor 1 (WT1) is an immunogenic antigen which is overexpressed in acute myeloid leukemia. Five elderly patients with acute myeloid leukemia were treated with WT1 recombinant protein in a phase 1/2 clinical trial (NCT01051063) and the majority of the patients achieved above-average response durations.^24^ MAGE family proteins are the most widely used immunogenic cancer-testis antigens with heterogeneous expression in tumor cells and poor understanding.^16,25^ A large-scale MAGE-A3 immunotherapy-focused phase 3 trial as an adjuvant therapy was conducted, however, the vaccine didn’t improve the clinical benefits of the patients with resected stage III melanoma, leading to its discontinuation.^26^ In a phase 3 trial, the MAGE-A3 protein vaccine was terminated because of its limited immunogenicity in patients with non-small cell lung cancer (NSCLC).^27^ Notably, it may be due to cross-reactivity with many MAGE-A isoforms. Therefore, a MAGE-A DNA vaccine consensus sequence was formulated to address this issue, which elicited a robust immune response, significantly leading to a substantial reduction in tumor growth in mice.^28^ Mucin 1 glycoprotein is widely distributed and abnormally glycosylated on cancer cells’ surfaces. Tecemotide, a peptide vaccine used against mucin 1, extended the overall survival (OS) in patients with advanced NCSLC who were concurrently undergoing chemoradiotherapy.^29,30^ Additionally, high expression of human epidermal growth factor receptor 2/neu (HER2/neu) makes some breast cancers highly malignant.^31^ The peptide vaccine Nelipepimut-S was safe in patients with HER2-positive breast cancer; however, no therapeutic effect was observed when compared to the placebo group.^32^ Nevertheless, the use of Nelipepimut-S plus trastuzumab resulted in specific and durable immune responses in patients with triple-negative breast cancer and resulted in a significant clinical benefit to the patients.^33^ A plasmid DNA vaccination encoding the HER2/neu intracellular domain was shown in a recent phase 1 clinical study to induce the production of antigen-specific type 1 T cells in a majority of patients with HER2-positive breast cancer.^15^

#### Viral antigen

Another approach involves targeting shared antigens from viral infections linked to certain cancer types. Epstein–Barr virus infection is associated with several cancers, including non-Hodgkin’s lymphoma and nasopharyngeal cancer.^34^ Several researches demonstrated that Epstein–Barr nuclear antigen 1, latent membrane protein (LMP) 1 and LMP2 could elicit antigen-specific T cells and induce favorable anti-tumor efficacy.^35,36^ With Epstein–Barr virus envelope proteins in a therapeutic Epstein–Barr virus cancer vaccine, the anti-tumor activity was further improved.^37^ Although vaccines for preventing HPV infection are currently available, a therapeutic HPV vaccine remains unexplored. Research has shown that an HPV16 RNA-lipid complex vaccine can induce the complete regression and establish durable T cell memory in rapidly progressing HPV-positive tumors.^38^ A therapeutic DNA vaccine, GX-188E, plus pembrolizumab, induced HPV E6 and E7 specific T cell immune responses and preliminary antitumor activity in patients with recurrent or advanced cervical cancer.^39^ Therefore, these studies indicate that viral antigens may be the optimal antigen targets option in virus-related cancers.

Preclinical and clinical researches indicate the generalizability of shared tumor antigens to serve as viable targets for cancer vaccination, although they are limited by tumor heterogeneity, poor immunogenicity, and immune tolerance. Therefore, further efficient approaches are under investigation to leverage the full range of tumor antigens.

### Neoantigen cancer vaccine

TAAs’ low expression in normal tissue results in “central thymic tolerance” within antigen-specific T cells leading to an inadequate stimulation in anti-tumor T cell immune responses.^40,41^ However, neoantigens derived from non-synonymous cell variants, including point mutations, gene fusions, and RNA editing events, are only expressed in tumor cells.^42–45^ Neoantigens can bypass thymus-negative selection because of the high immunogenicity of somatic tumor mutation-acquired neoantigens, leading to a robust neoantigen-specific T cell response.^46^ Genomic and transcriptional profiling has made identifying putative neoantigens possible in cancers that have high immunogenicity, with advancements in next-generation sequencing and bioinformatics.^13,47^ To date, it is noteworthy that current researches suggest that only a minority of neoantigens have the capacity to induce neoantigen-specific T cell responses, making neoantigen prediction critical for clinical success.

#### Neoantigen identification

**Fig. 3:**
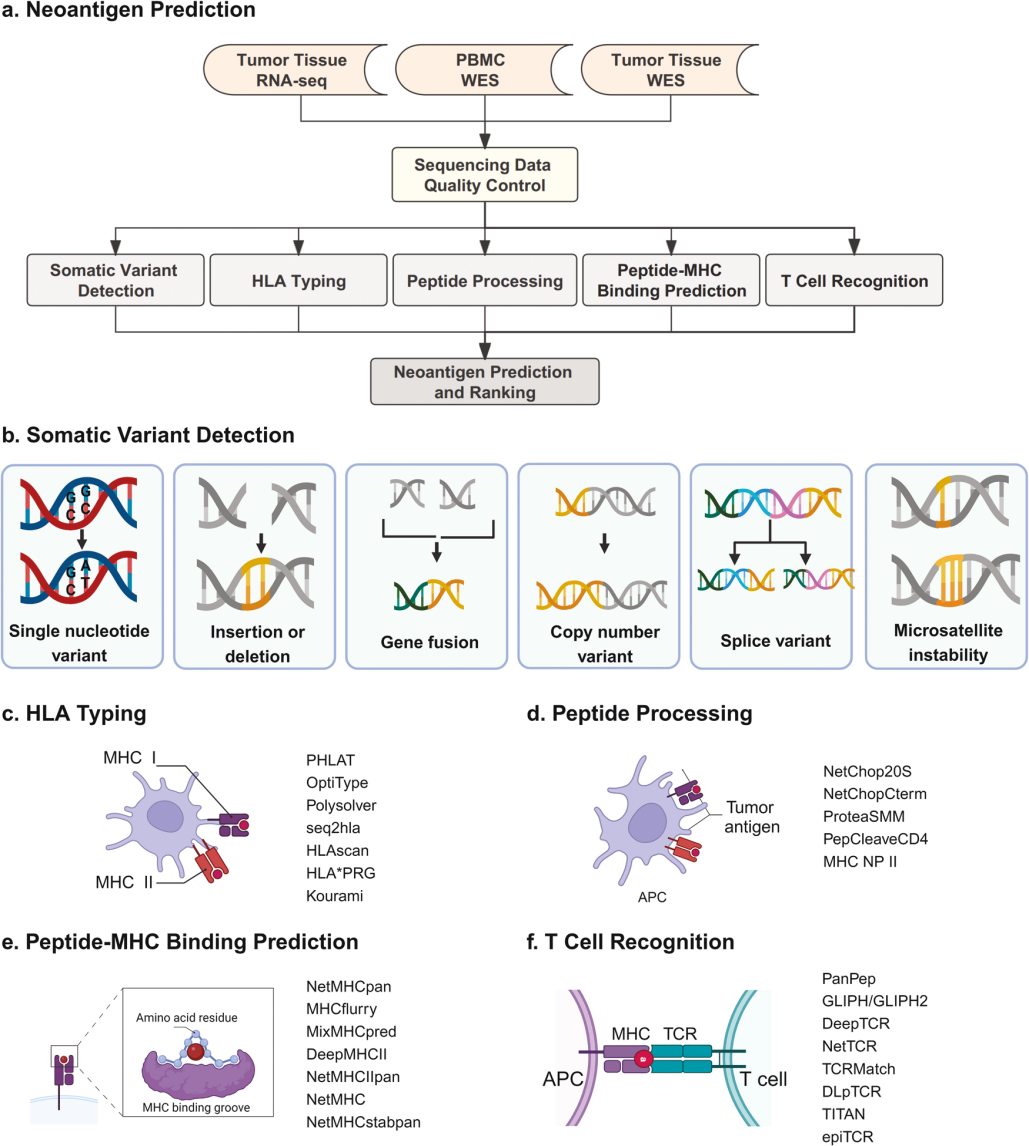
Prediction of neoantigen candidates. **a** An overview of the bioinformatic characterization of neoantigens. **b** The somatic mutants originate from multiple sources, including single nucleotide variants, insertions/deletions, gene fusion, copy number variant, splice variant and microsatellite instability. **c** HLA typing from WES and RNA-seq could be found by the in-silico tools. **d** Peptide processing prediction with several algorithms. **e** Available bioinformatic pipelines for peptide-MHC binding prediction. **f** Available bioinformatic pipelines for T cell recognition. RNA-seq, RNA-sequencing; WES Whole exome sequencing, PBMC Peripheral blood mononuclear cell, HLA Human leukocyte antigen, MHC Major histocompatibility complex, APC Antigen presentation cell, TCR T cell receptor

Neoantigens are categorized according to the somatic mutations type that cause non-synonymous protein changes (Fig. 3a).^48^ The most potential neoantigen sources in cancer can be found in Fig. 3b. The identification of immunogenic neoantigens has significantly benefited from the development of in silico methods and tools that utilize high-throughput sequencing data.^49^ Recent investigations have conducted thorough characterizations of neoantigens that originate from single nucleotide variants (SNVs) and small insertions and deletions (INDELs).^49^ Moreover, mutations stemming from gene fusions, copy number variations, transcriptional variants (such as selective splicing, promoter, and A-to-I editing), and microsatellite instability have been shown to give rise to neoantigens. Traditional complementary DNA library screening methods are confined to the identification of variant antigens in specific transcripts, especially GC-rich or low-expression transcripts. However, whole-exome sequencing (WES) and mass spectrometry (MS) have emerged as potent approaches for identifying HLA-bound peptides and forecasting distinctive cancer epitopes to facilitate personalized vaccine development.^50–52^ Distinct high-throughput sequencing data are required for the identification of neoantigens originating from different sources. In the case of SNV-based sources, pTuneos can evaluate actual immunogenicity by considering natural processing and presentation, utilizing data from tumor-infiltrating lymphocyte-recognized neopeptides in a melanoma cancer vaccine cohort.^53^ Several integrated processing tools like pVAC-seq,^54^ TIminer^55^ and ProGeo-Neo^56^ can pinpoint neoantigens targeting SNV and INDEL sources. These tools utilize input from WES/RNA-sequencing (RNA-seq) data and implement a series of filtering steps guided by predefined threshold values to eliminate potential false positives. In situations involving gene fusions, the automated INTEGRATE-Neo pipeline stands as the pioneering platform for gene fusion neoantigen discovery.^57^

Once candidate neoantigens are identified, it becomes crucial to verify their capability for effective MHC molecular presentation and recognition by TCRs. Consequently, substantial effort is still warranted to access the potential immunogenicity of neoantigen candidates. This aspect will be examined in detail in the subsequent section.

##### HLA typing

The presentation of neoantigens necessitates specialized APCs with MHC I or II molecules. Given the extensive polymorphism of human HLA alleles—comprising over 24,000 distinct HLA gene complexes—accurate HLA typing is imperative to precisely predict neoantigens.^58^ Calculated HLA typing can be accomplished by analyzing patient peripheral blood samples through the NGS platform or sequence-specific PCR amplification. Recently developed HLA typing algorithms, such as OptiType,^59^ Polysolver,^60^ HLAscan,^61^ and PHLAT^62^ are effective for the identifying HLA class I alleles, demonstrating a high degree of precision. While benchmarking studies for HLA class II algorithms alone are comparatively limited, those that have been conducted indicate that combined algorithms targeting both HLA class I and II, like seq2hla^63^ and HLA*PRG,^64^ exhibit high accuracy rates. Kourami, a graph-guided classical HLA gene assembly technique, empowers the construction of allelic sequences using high-coverage whole genome sequencing data.^65^ Successful neoantigen delivery is the initial stride in generating tumor-specific T cells, rendering the deletion or reduced expression of HLA gene loci a pivotal mechanism for evading immunotherapy.^66^ An instance involving a patient with a tumor and poly-neo-epitope-specific immunity, who received a personalized cancer vaccine, highlighted the discovery of β2-microglobulin transcript deletions in HLA class I transcription analysis through NGS sequencing. This patient subsequently encountered resistance to the tumor vaccine and experienced disease recurrence.^67^ The reduction in the expression of the transporter associated with antigen processing (TAP) has also been recognized as a factor that hinders the presentation of tumor antigens.^68^ Thus, discerning the HLA locus while concurrently elucidating the dynamics of related presenting genes empowers investigators to discover neoantigens binding to expressed and unmutated alleles.

##### Peptide processing

To function as a natural T cell antigen, the original peptide must undergo a series of processing steps. These processes are essential to prepare the peptide for presentation on the MHC molecule. The natural processing and presentation of antigens constitute an intricate process, necessitating precise peptide processing to enable its effective presentation on the MHC molecule. Thus, even if a peptide is predicted to exhibit a strong binding affinity to MHC, it may not trigger a T cell response due to upstream peptide processing factors. These factors encompass how the protein is cleaved, trimmed, loaded onto the MHC molecule, and transported to the cell surface, which prevents the actual loading of the peptide.^69^ Owing to constraints posed by the immune proteasome, not all k-mer peptides can be naturally generated in vivo, and only a fraction of these peptides can be translocated to the appropriate cellular compartments to interact with MHC molecules.^70^ As a result, several computational tools have been developed with a specific focus on immune proteasomal processing and peptide cleavage. A pivotal step preceding the peptide-MHC interaction is proteolysis, the breakdown of proteins into peptides facilitated by the immune proteasome.^71^ Methods like ProteaSMM and NetChop 20 S were most effective in capturing in vitro proteasome digestion patterns for MHC class I antigens. Conversely, NetChop Cterm trained on MHC-I ligand data demonstrated superior predictive capabilities for in vivo whole-cell proteolysis.^71^ Moreover, TAP proteins play a role in translocating peptide fragments from the cytoplasm into the endoplasmic reticulum, thereby facilitating the process of loading these peptides onto MHC molecules. Tools targeting TAP protein affinity, such as TAP-hunter, have been devised to predict peptide transportation efficiency.^72^ The endeavor to create an integrated process encompassing relevant metadata and accessing data on naturally processed peptides from an immunological perspective is pivotal to identify optimal target antigens and predict class I and II epitopes.^73^

##### Peptide-MHC binding prediction

The algorithms development specifically designed to accurately predict the binding of neoantigens to MHC class I and class II molecules is a crucial approach in forecasting the immunogenicity potential of neoantigens. Here, we present the main algorithmic innovations and the data classes used to train these algorithms. Established tools used for predicting MHC binding affinity are trained using data derived from in vivo binding affinity measurements or eluting ligands detected by MS.^74^ By directly studying ligands eluting from peptide-MHC (pMHC) complexes, predictive models can encompass distinctive characteristics of peptides that have undergone the complete processing pathway. A systematic benchmarking of pMHC class I combined with predictors revealed that NetMHCpan and MHCflurry exhibited the best area under the receiver operating characteristic curve.^74–76^ Benchmarking analysis demonstrated that MixMHCpred 2.0.1 excelled predicting peptide binding to the HLA-I isoforms by evaluating the probability of peptide sequence to be presented on the cell surface.^77^ While algorithms targeting the binding affinity to MHC class II are less mature, a blend of matrix-based techniques and artificial networks, exemplified by DeepMHCII and NetMHCIIpan, facilitates the accurate recognition of CD4^+^ T cell epitopes.^78,79^ Moreover, the stability of pMHC complexes can effectively increase the likelihood of recognition by T cells and is a better correlate of immunogenicity compared to MHC class I binding affinity.^80^ Therefore, researchers developed a neural network-based peptide-MHC-I complex stability pan-specificity predictor (NetMHCstabpan), which, in combination with an MHC binding predictor, can significantly improve the prediction of antigenic epitopes.^81^ To ascertain the thermal stability of cleavable peptide/HLA complexes, the NetMHC 4.0 approach can also be employed, facilitating the screening of mutant peptides with the utmost likelihood of cell surface expression.^82^

##### T cell recognition

Precise prediction and identification of interactions between TCR and pMHC complexes constitute a substantial computational challenge within the realm of therapeutic cancer vaccines. A range of computational tools have emerged to analyze diverse TCR patterns and forecast peptide-TCR-specific interactions. GLIPH,^83^ DeepTCR^84^ and TCRmatch^85^ establish global similarities and shared specificities among TCR sequences. NetTCR has been formulated by constructing a peptide-specific approach.^86^ Early iterations of these tools could solely discern binding patterns of antigens based on numerous established TCR binding profiles, yet they fell short in recognizing antigens that had never surfaced within the body or those only scant TCR binding profiles were available.^87,88^ In this context, TITAN has enabled the exploration of generalization capabilities for unencountered TCRs and/or epitopes, employing bimodal neural networks that explicitly encode TCR sequences and epitopes.^89^ Given the extensive diversity intrinsic to the TCR interaction landscape, there remains considerable room for refining the learning of peptide-TCR binding prediction, especially for peptides not represented within the training dataset or for exogenous peptides. PanPep is a general framework that identifies TCR-antigen binding in 3D crystal structures, amalgamating the principles of neural Turing machines and meta-learning.^90^

Furthermore, there are still some assessments of immunogenicity of neoantigen epitopes that need to be taken into account, including transcript expression, dissimilarity to self, similarity to epitopes associated with pathogens, mutation clonality and indispensability and loss of heterozygosity of essential gene product, which can facilitate the selection of neoantigen candidates that have the potential to generate T cell responses, contributing to the subsequent customization of personalized vaccines for individual patients.^48,49^ Accurately identifying and selecting biological knowledge-guided and computational algorithm-assisted immunogenic neoantigen candidates is the cornerstone of personalized tumor vaccines’ clinical success. Many groups have developed proprietary and unique algorithms for selecting immunogenic epitopes that could help drive the next generation of cancer immunotherapies and personalized cancer vaccines.^90–93^

#### Neoantigen-based cancer vaccine

**Fig. 4:**
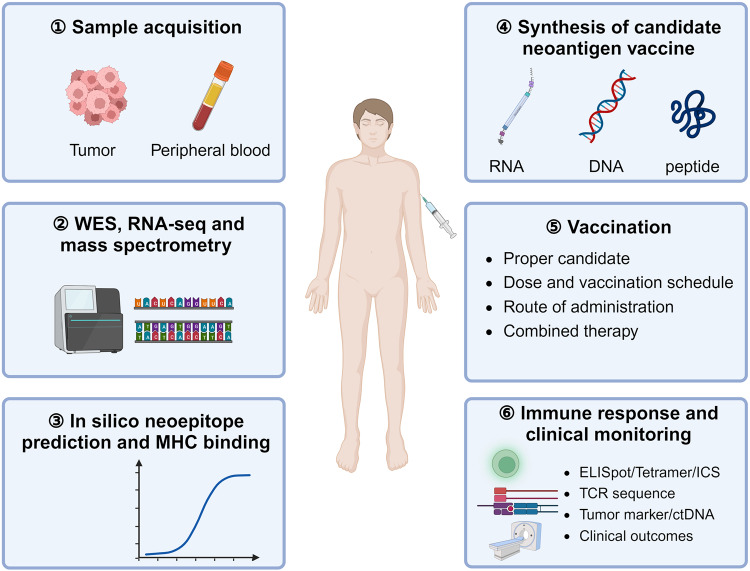
Overview of the factors involved in the different steps from the neoantigen cancer vaccine preparation to application. Following the tumor biopsy, deep sequencing was performed on tissue and blood samples to identify immunogenic neoantigens. A neoantigen-based cancer vaccine was subsequently developed using an appropriate design strategy and administered to the patient, with regular monitoring of the immune response and clinical outcomes. WES Whole exome sequencing, RNA-seq RNA-sequencing, MHC Major histocompatibility complex, ELISpot Interferon-γ Enzyme-Linked Immuno-Spot, ICS Intracellular cytokine staining

Neoantigen vaccines are promising for stimulating cytotoxic T cells to mount effective anti-tumor responses (Fig. 4). In 2014–2015, several teams successively identified neoantigens using MS and WES/RNA-seq and effectively treated patients with metastatic cholangiocarcinoma and advanced melanoma, establishing a foundational framework for the development of personalized cancer immunotherapy.^50,94–96^ The first-in-human application of an mRNA-based neoantigen vaccine effectively inhibited melanoma recurrence, resulting in sustained progress-free survival (PFS).^67^ Similarly, an early study on a personalized cancer vaccine targeting 20 predicted neo-epitopes with high HLA molecular-binding affinity showed that immunization induced polyfunctional antigen-specific T cells in patients with high-risk melanoma.^40^ Subsequently, several recent studies have demonstrated their immunogenicity with favorable clinical outcomes, particularly with the help of ICI. NEO-PV-01, a long peptide cancer vaccine comprising up to 20 neoantigens combined with Nivolumab, induced the cytotoxic neoantigen-specific T cells in patients with NSCLC, melanoma, or bladder cancer. These activated T cells were responsible for initiating the destruction of tumor cells.^97^ Recently, an investigational personalized mRNA cancer vaccine mRNA was reported to significantly reduce the melanoma recurrence risk or death by 44% combined with pembrolizumab, and the combination did not significantly increase the risk of severe side effects, which has been granted breakthrough therapy designation by FDA.^98^ Related studies have shown that although patients experience relapse post-vaccination, lesion progression can be controlled using ICI, suggesting a synergistic effect between neoantigen vaccination and ICI treatment.

Patients with minimal residual disease are less affected by production pipeline timeline and the immune mechanisms of suppressive tumors have not yet been fully established, perhaps making them more suitable for vaccine application. Designing an optimal delivery platform, selecting the optimal combination therapy, avoiding immune escape and eliciting a robust T cell immune response remains challenging, and these will be comprehensively addressed in the following review.

## Vaccine constituent and platform

Precisely delivering antigens to their intended sites presents a significant obstacle to effectively developing cancer vaccines. Multiple factors must be considered when selecting an appropriate platform for cancer vaccines, including components and delivery methods. Established delivery methods include DNA, RNA, and peptide vaccines, while newer platforms are being investigated. This section discusses the advantages and limitations of each platform in order to determine the most effective neoantigen-based cancer vaccine delivery system (Table 1).

**Table 1:** Advantages and disadvantages of different forms in neoantigen cancer vaccine

Vaccine types	Advantages	Disadvantages
DNA vaccine	● Low cost; ● Cell-independent production; ● Long-lasting immune response ● potential for targeting multiple neoantigens	● Risk of integration into host genome; ● Risk of autoimmune reactions ● Low transfection efficiency
RNA vaccine	● Rapid development and easy modification; ● High immunogenicity; ● Cell-independent production; ● Intrinsic adjuvant effect; ● High efficiency into DCs	● Fast degradation speed; ● Potential for inflammatory reaction
Peptide vaccine	● High specificity and safety; ● Cell-independent production; ● Low risk of autoimmunity; ● Direct presentation on MHC in short peptides; ● Proven clinical activity in SLP	● High cost; ● Complex manufacture; ● Requirement for suitable adjuvants; ● Potential for HLA-restriction
Cell-based vaccine	● Strong immune stimulation; ● Multi-form antigen loading	● High cost; ● Potential for immunogenicity of the cells; ● Need for patient-specific customization
Viral and bacterial vector vaccine	● High immunogenicity; ● Long-lasting immune response; ● Self adjuvanticity	● Potential for vector immunogenicity; ● Need for specialized storage conditions

### DNA vaccine

DNA cancer vaccines are attractive because their efficient manufacturing process. DNA vaccines can simultaneously deliver multiple antigens in the same construct at the same time. The DNA cancer vaccine GX-188E, designed to target HPV-16/HPV-18 E6 and E7, was administrated to patients diagnosed with HPV-positive advanced cervical cancer and displayed a favorable safety profile. Objective remission was achieved in 19 of 60 patients [overall response rate (ORR): 31.7%], among whom 6/60 had complete remission (CR) and 13/60 had partial remission (PR), respectively, showing considerable therapeutic efficacy.^39^ In a phase 1 non-randomized clinical trial involving 66 patients with advanced HER2/neu-positive breast cancer, a plasmid DNA vaccine encoding the intracellular domain of HER2/neu elicited an antigen-specific T cell response that persisted even after the vaccination was completed.^15^ Numerous preclinical studies have explored the potential of DNA vaccines to enhance the antigen-specific immune response. One innovative approach involves DNA nanodevice vaccines, which are designed to assemble antigen peptides and adjuvants within tumor cells, leading to a potent and enduring T cell response.^99^

DNA vaccine based on plasmids can encode antigens and other immunostimulatory cytokines, including granulocyte-macrophage colony-stimulating factor (GM-CSF) and interleukin-2 (IL-2). Cytosolic receptors can recognize double-stranded DNA structures, allowing plasmid DNA vaccines to simultaneously activate innate immunity.^100^ However, many limitations still exit in the DNA vaccine clinical application. DNA must conquer both extracellular and intracellular barriers to migrate into the cell nucleus, posing a challenge for DNA-based antigen delivery system. Physical methods are the most common administration strategies, including electroporation, gene gun and sonoporation, which lead to the difficulties in clinical promotion.^101–103^ DNA vaccines have demonstrated limited immunogenicity in clinical trials, despite their effective delivery. Efforts have been made to enhance the efficacy of DNA vaccines through various strategies. These include advancements in the design of DNA vaccine vectors, the incorporation of cytokine adjuvants, and the exploration of innovative non-mechanical delivery methods.^104^

### RNA vaccine

The emergency use of two mRNA COVID-19 vaccines brought mRNA vaccines back into the spotlight. The MIT Technology Review released a compilation of the top 10 breakthrough technologies for 2021, with mRNA vaccines ranking first due to their dramatic changes in revolutionizing the medical field.^105^ Compared to the risk of integration into the host genome in DNA vaccines, mRNA is produced using in vitro transcription and can be directly translated into protein once they enter the cytoplasm, offering a well-tolerated delivery method without the risk of genome integration.^106^ The mRNA is also transiently expressed in cells, enabling repeated inoculations.^107^ Furthermore, mRNA-encoded units are flexible and versatile, making it feasible to encode tumor antigens and immunomodulatory molecules. This flexibility is valuable for inducing both adaptive and innate immunity effectively.

Technical barriers to mRNA vaccines are centered on their molecular design and in vivo delivery efficiency. mRNA modification and the sequence design of the its regulatory and coding regions play a crucial role in determining mRNA stability and translation efficiency. The antigen translation efficiency can be enhanced using codons preferred by somatic cells to limit the GC content of mRNA sequences and calculating and selecting mRNA sequences with high codon adaptation indices. mRNA stability is enhanced by optimizing the secondary structure of mRNAs and calculating and selecting mRNA sequences with high minimum free energies while guaranteeing the codon adaptation indices.^108–110^ mRNA is a negatively charged biomolecule that enter the cell through the negatively charged cell membrane to achieve therapeutic effects. Therefore, to reduce the extracellular degradation of naked mRNA by RNA enzymes, several mRNA delivery systems have been designed to lengthen the mRNA circulation time in vivo, improve translation efficiency, and increase antigens uptake by APCs.^111^ Positively charged cationic liposomes binding to negatively charged mRNA contributes to APC endocytosis. Protamine is a polycationic natural peptide with a positive charge that can bind to mRNA to form complexes and maintain mRNA stability.^112^ The self-adjuvanted mRNA CV9103 coated with protamine, encoding several prostate cancer-specific TAAs, was safe in patients with prostate cancer, although no clinical benefit was observed in phase 1/2 clinical studies.^113^

Several studies have been carried out to access the anti-tumor efficacy of LNP-formulated mRNA neoantigen cancer vaccines. Neoantigens and driver gene mutations were tandemly linked into a single mRNA sequence, coated with LNP, and administered to patients with gastrointestinal cancer. The cancer vaccine could elicit a robust and broad neoantigen-specific immune response. TCRs targeting KRAS G12D have been isolated and identified from patients’ blood after tumor vaccine application.^114^ The latest clinical trial of mRNA-4157 in high-risk melanoma showed that the combination with mRNA-4157 and anti-programmed cell death protein 1 (PD-1) greatly prolonged the distant-metastasis free survival and reduced the risk of developing distant metastases or death by 65% compared to pembrolizumab alone. Owing to these exciting clinical findings, mRNA-4157 is poised to become the world’s first mRNA personalized tumor vaccine to undergo phase 3 clinical study.^98^ Notably, after the first patient was dosed with a vaccine using non-nucleoside modified RNA for personalized cancer therapies in 2012, BioNTech developed a new class of versatile, tailored mRNA therapeutics using multiple mRNA formats with distinct properties capable of addressing several cancers. Several pipelines targeting TAAs have been developed for NSCLC, HPV-related cancers, melanoma and prostate cancer.^22,38^ The individualized mRNA neoantigen cancer vaccine BNT122 is being examined in a randomized phase 2 trial as a first line treatment combined pembrolizumab in patients with untreated melanoma, after a success in phase 1 clinical trial.^115^ Immunization of BNT122 in surgically resected pancreatic ductal adenocarcinoma (PDAC) induced high-intensity neoantigen-specific T cells production in vivo and effectively prolonged recurrence-free survival (RFS) in patients who had immune response.^116^ Besides, BioNTech is launching a U.K. trial of personalized mRNA cancer vaccines since September 2023 and it aims to deliver 10,000 Mrna cancer vaccines to cancer patients before 2030.^117^ Given these outstanding clinical outcomes, personalized mRNA cancer vaccine is hopefully authorized by 2030.

Clinical trials have demonstrated that appropriate mRNA structure, stability, and delivery methods can enhance anti-tumor immunity, however, further clinical benefits require confirmation through rigorous trials.

### Peptide vaccine

**Fig. 5:**
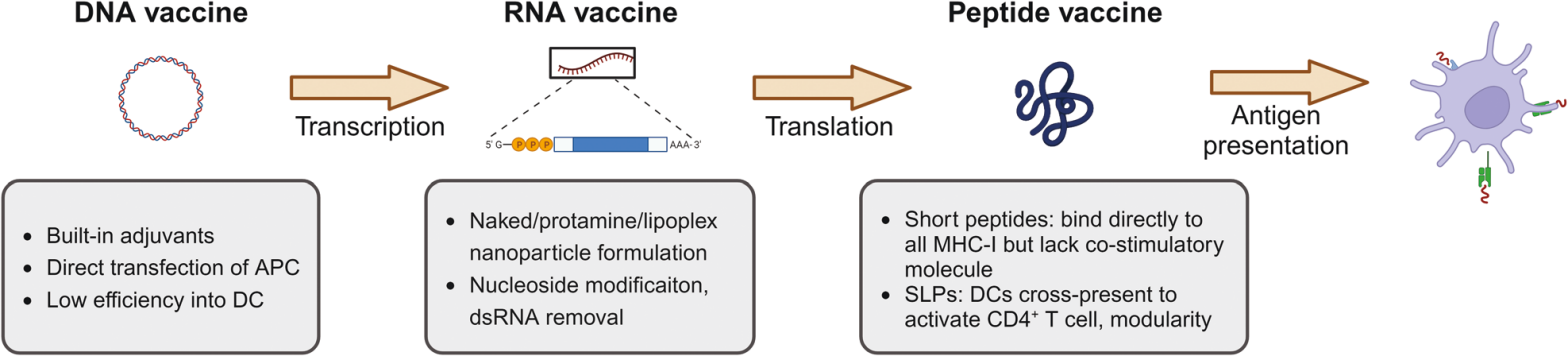
Direct delivery of antigen. TAA or TSA, which are the antigens with tumor immunogenicity, could be delivered directly into the body as DNA, RNA, or peptides using different adjuvants. DNA and RNA vaccines provide the potential for more efficient delivery and sustained expression of the target antigen, while peptide vaccines are generally easier to produce and have a strong safety profile. TAA Tumor-associated antigen, TSA Tumor-specific antigen, APC Antigen presentation cell, DC Dendritic cell, MHC-I Major histocompatibility complex-I, SLPs Synthetic long peptides; dsRNA, Double-stranded RNA

Peptide-based cancer vaccines could provide many advantages, including high specificity and safety, insusceptibility to pathogen contamination and a low risk of autoimmunity. Vaccines containing 8–12 amino acids derived from tumor antigens include MHC-I-binding short peptides preferably endocytosed, processed, and presented by professional APCs to elicit peptide-specific T cells.^118^ Synthetic long peptide (SLP) vaccines typically contain 25–35 amino acids, frequently encompassing multiple epitopes or larger portions of the target protein.^119^ Therefore, SLP vaccines containing multiple epitopes can elicit broader and more diverse immune responses using longer peptide sequences. This broader response may enhance vaccine effectiveness by targeting a wider range of antigens or strains.^120^ Utilizing SLPs rather than short peptides can further enhance peptide stability and antigen delivery efficiency (Fig. 5).^121^ However, SLP cancer vaccines have disadvantages such as complex preparation, potential for HLA-restriction, and rapid degradation. Therefore, developing more effective immune formulations is necessary for enhancing neoantigen-derived peptide-specific immunity.

Tumor antigens involved in SLP vaccines may have suboptimal immunogenicity, indicating that they may not induce a robust immune response in all individuals. Enhancing the immunogenicity of SLP vaccines is an area for improvement. Immune stimulation adjuvants have been used to enhance co-stimulatory signals to promote local immune cell proliferation in the tumor.^122^ GM-CSF is a strong adjuvant that can improve T cell priming efficiency by recruiting dendritic cells (DCs) into the skin after vaccination.^123^ However, a phase 3 clinical trial that investigated the combination of GM-CSF plus peptide vaccination did not show a significant improvement in recurrence-free survival (RFS) or OS for patients with advanced melanoma.^124^ The SLP vaccine can induce a robust T cell response with immunomodulatory adjuvants, including polyriboinosinic-polyribocytidylic acid-poly-L-lysine carboxymethylcellulose (poly-ICLC), cytosine-phosphate-guanine (CPG) and Toll like receptor (TLR). In a phase 1 clinical trial targeting patients with glioblastoma, an innovative strategy involving a vaccine from a pool of pre-manufactured unmutated antigens (APVAC1) followed by immunization with a vaccine derived from neoantigens (APVAC2) elicited a sustained response of central memory CD8^+^ T cells and predominantly CD4^+^ T cell responses, which was characterized by multifunctionality and T-helper (Th)1 polarization.^125^ TLRs are important components of innate immune system, whose agonists expressed in intracellular compartments can trigger potent antiviral and anti-tumor immune responses. Boosting innate immunity through TLR7 or TLR8 are effective antiviral strategy.^126^ In a study of patients with resected locally advanced melanoma, the SLP vaccine LPV7 containing seven TAA peptides in combination with incomplete Freund’s adjuvant (IFA) and agonists for TLR3 (poly-ICLC) and TLR7/8 (resiquimod) induced TAA-specific T cells, but did not enhance the immune response rate compared to the vaccine alone, indicating that immunogenicity may be enhanced by poly-ICLC plus IFA.^127^ Although a few clinical studies have investigated different tactics, a good ORR has not been achieved in patients with cancer. Additionally, SLP vaccines may be restricted to individuals with specific HLA types, limiting their universal applicability.^128^ Expanding the range of HLA-restricted SLP vaccines or developing strategies to overcome HLA restrictions could increase the number of individuals who will benefit from this cancer vaccine type.

Notably, the SLP vaccine research is evolving continuously, and ongoing studies and technological advancements may further improve their design, formulation, and applications.

### Cell-based vaccine

Cell-based cancer vaccine is a cancer vaccine that utilizes patient’s own immune cells. DCs are harnessed to both antigens soluble and particulate exogenous states in diverse formats, encompassing DNA, RNA, proteins, peptides, or tumor lysates. To augment the antigenicity of vaccines, various carriers such as emulsions, liposomes, and polymeric particulate carriers are employed.^129^ This uptake is facilitated through techniques like electroporation or lentiviral transduction.^130–134^ Besides conventional APCs’ function, isolated DCs are antigen donor cells, transferring to endogenous cross-presenting APCs with multiple layers of co-stimulation and can secrete key cytokines.^135^ The first individual DC cancer vaccine was administrated in 6 patients diagnosed with melanoma. By immunized with a personalized neoantigen pulsed autologous DC vaccine in patients with metastatic lung cancer, peripheral blood mononuclear cells (PBMC) responded strongly to predicted neoantigens, thus expanding the diversity of alternative therapies for lung cancer.^136^ In a recent phase 3 clinical trial, an autologous tumor lysate-loaded DC vaccine (DCVax-L) was incorporated into the standard treatment regimen for both newly diagnosed glioblastoma (nGBM) and recurrent glioblastoma (rGBM). The results revealed that patients with nGBM who received DCVax-L had a median OS of 19.3 months, significantly longer than the 16.5 months observed in control groups (HR = 0.80; 98% CI, 0.00–0.94; *p* = 0.002), demonstrating the substantial extension in long-term survival of patients with nGBM due to DCVax-L. The study also showed that the median OS for 64 patients with rGBM who received DCVax-L was 13.2 months, compared to 7.8 months for those without DCVax-L treatment. These results from the phase 3 clinical trial offer a newfound sense of hope for the treatment of patients with glioblastoma.^137^

Optimizing the methods for generating and manipulating DCs ex vivo can improve their maturation, activation, and antigen-presenting capabilities, leading to a more potent immune response. Early DC-based cancer vaccines mainly used PBMC from patients for in vitro isolation and culture.^138^ However, PBMC-derived DCs have the limitations that not possess all the co-stimulatory molecules and antigen cross-presentation mechanisms available to other DC subpopulations. DC precursor cells in bone marrow are categorized into plasma cell-like DCs (pDCs) and conventional DCs (cDCs).^139^ pDCs play a key role in secreting type I interferon along with the antigen presentation.^140^ cDC1s can cross-present and induce cytotoxic CD8^+^ T cell immune responses, whereas cDC2s are specialized in presenting soluble antigens, particularly to CD4^+^ T cells.^141^ Therefore, efforts to optimize in vitro culture to induce high-quality required DC subsets become the main challenge for future DC vaccines development. Technologies for hematopoietic stem and progenitor cells-derived and cord blood monocyte-derived DC subsets are undergoing preclinical investigation.^142,143^

Enhancing DC vaccines’ lymphoid-homing ability can improve their effectiveness by ensuring optimal migration of DCs into lymphoid tissues, where they can interact with and immune cells, respectively. Chemokine receptors contribute crucially to directing DC migration into lymphoid tissues. C-C chemokine receptor (CCR) 7 mediates mature DCs’ lymphoid homing by binding with C-C motif chemokine ligand (CCL) 21/19 ligands on the surface of lymphatic endothelial cells, upregulating CCR7 expression on DCs, thereby promoting their migration into the lymph nodes.^144^ A hybrid nanovaccine exhibited high expression of CCR7 on DC membrane vesicles and enhanced the efficiency of lymph node targeting.^145^ Factors such as TLR agonists, cytokines, or immune stimulatory molecules, can promote DC maturation and activation to further promote DC lymphoid homing.^146,147^ Immunogenic cell death induces human neutrophil elastase and the TLR3 agonist hiltonol plus breast cancer exosomes to form a DC vaccine that adequately exposed tumor antigens following cDC1 activation in situ and tumor-reactive CD8^+^ T cell cross-priming.^148^ Delivering DC vaccines through specific routes that facilitate their migration to lymphoid tissues, such as intradermal or subcutaneous injections, can enhance their lymphoid homing ability compared to intravenous administration.^149^ DC vaccines can augment the immune response and improve tumor control with the help of ICI or adoptive T cells.^150,151^

Numerous antigen peptide-DC vaccines are currently under development, and selecting peptides in the pool and their grouping for optimal efficacy should be given more attention. Customizing the neoantigen peptide pool is important to cover t tumor heterogeneity and enhance multiple tumor-specific target recognition. When selecting antigens for vaccination, it’s crucial to consider competition for antigen recognition at the surface of APCs and the affinity of the selected epitopes.^152^ After immunization with complex antigens, a common observation is a preferential induction of immune responses against immunodominant epitopes.^153^ Furthermore, the presence of high variant allele frequency and peptides with strong HLA-binding affinity can impact the efficacy of cytotoxic T cell- mediated immunity.^47^ Successful eradication of tumors necessitates a coordinated response involving both CD4^+^ and CD8^+^ T cells, although CD8^+^ T cell responses are conventionally regarded as pivotal in the context of anti-tumor immunity.^154^ DCs activated with nucleic acid or protein containing multiple antigenic epitopes can trigger MHC class I- and II-biased immune responses, leading to a diverse repertoire of both CD4^+^ and CD8^+^ T cell responses.^155^ The inclusion of a Th epitope can augment DC maturation and the activation of Th cells, further enhancing anti-tumor activity in vivo.^156^ Further researches and clinical trials should optimize DC vaccines’ immune response intensity and improve their therapeutic outcomes.

### Viral and bacterial vector vaccine

Viruses and bacteria possess inherent immunogenicity, and their genetic material can be modified to incorporate cancer antigens. Recombinant viruses can serve as vectors to infiltrate immune cells and deliver substantial quantities of tumor antigens, which consequently trigger the activation of T cells and instigate the initiation of anti-tumor immune responses.^157,158^ While preclinical studies have shown promise, clinical trials utilizing viral vectors as standalone cancer vaccines have, for the most part, yielded disappointing results.^158^ Nevertheless, clinical outcomes can be improved when they are combined with other therapies. A phase 2 study showed that T-VEC, an engineered oncolytic herpes simplex 1 virus encoding GM-CSF, combined with standard neoadjuvant therapy in patients with triple negative breast cancer, achieved complete pathologic response in 45.9% of the patients, 89% remained disease-free 2 years post-treatment, and most patients had higher levels of anti-tumor T cells and immune signaling pathways activation.^159^ Similarly, TG4010, which is a recombinant modified cowpox virus encoding MUC1 and IL-2, in conjunction with chemotherapy in advanced NSCLC or PANVAC plus docetaxel for patients with metastatic breast cancer clearly offers additional clinical advantages for patients.^160,161^

Over the past century, Busch and Fehleisen et al. first reported a link between cancer and bacteria and found that *Streptococcus pyogenes* infection could lead to tumor regression.^162^ Currently, using bacteria as carriers to deliver tumor antigens has many advantages. Bacillus is highly active under prevailing anaerobic/hypoxic conditions [mostly hypoxic tumor microenvironment (TME)].^163^ Some auxotrophic strains, such as Salmonella, are attracted to the TME to absorb metabolic nutrients.^164^ Summarily, bacteria have several advantages as potential carriers for anti-cancer drugs or gene vaccine delivery. They thrive in a hypoxic TME, are attracted to the tumor for nutrient uptake, and migrate into remote areas of the tumor. These characteristics make them promising candidates for anti-cancer treatment.

### Novel platform for delivery system

Different nano-drug delivery systems are currently being developed with the aim of enhancing the precision targeting of cancer vaccines and improving the uptake of neoantigens by DCs.^165–168^ These systems include LNPs, exosomes, bacteria-derived outer membrane vesicles, and amphiphilic vaccines. One promising approach is personalized DC-mimicking nanovaccine, including the nanoDC vaccine, using components from *Escherichia coli* and tumor cells. It efficiently delivered TAAs and induced the maturation in bone marrow-derived cells through the stimulatory of the interferon genes signaling pathway. It also exhibited remarkable lymph node homing ability and elicited strong TAA-specific T cell responses in mice.^169^ Another way involves the use of lipopolyplex vector, which consists of a bilayer structure with polymer- and phospholipid-encapsulated mRNA forming the inner core and outer layer, respectively.^170,171^ A phase 1 trial (NCT05198752) was conducted involving patients with advanced solid tumors to explore the lipopolyplex-mRNA vaccine’s safety and immunogenicity.

Overall, developing these nano-drug delivery systems holds great promise for enhancing the efficacy of cancer vaccines by improving the delivery efficiency of neoantigens to DC cells in lymph nodes. These approaches have the potential to significantly enhance the generation of antigen-specific T cell responses, which could ultimately improve clinical outcomes for patients with solid tumors.

## Clinical trial landscape

Clinical studies of cancer vaccines are mainly conducted in patients with advanced solid tumors, with safety, immunogenicity and clinical benefits as the endpoints. As of July 2023, 1847 clinical trials have been registered on the United States National Library of Medicine’s ClinicalTrials.gov website with the keyword “cancer vaccine”, of which 947 have been completed, and 224 are actively recruiting. Neoantigen-based cancer vaccine research accounts for only a small portion of the research. Table 2 presents the representative relevant clinical studies. Current findings indicate that the use of cancer vaccines alone does not yield effective outcomes in prolonged patient survival, although antigen-specific T cell immune responses have been detected in most clinical trials. Therefore, enhancing T cell activation and anti-tumor efficacy is the most significant therapeutic challenge.

**Table 2:** Selected ongoing clinical trials of therapeutic cancer vaccines

Clinical trial	Target antigen	Platform	Adjuvant	Dose and schedule	Indication	Combination therapy	Administration route	Primary Endpoint	Enrollment	Investigators	Start year
**mRNA cancer vaccine**											
NCT05933577 (phase 3)	Neoantigen mRNA	LNP	/	1000 μg,9 doses, q3w	High-risk melanoma	Pembrolizumab	Intramuscular injection	RFS	1089	Merck Sharp & Dohme LLC, ModernaTX, Inc.	2023
NCT05886439 (phase 1b/2a)	Neoantigen mRNA	Autologous DC	/	4 priming followed by 3 boosters	Incurable lung cancer	Pembrolizumab or durvalumab	Not mentioned	DLT, AE	40	Chinese Academy of Medical Sciences	2023
NCT05198752 (phase 1)	Neoantigen mRNA	Lipopolyplex	/	50–150 μg, q3w	Multiple solid tumors	/	Subcutaneous injection	DLT	30	Stemirna Therapeutics	2022
NCT04486378 (phase 2)	Neoantigen mRNA	LNP	/	qw, q8w, up to 12 months	Resected Stage II (High Risk) and Stage III Colorectal Cancer	Pembrolizumab	Intravenous injection	DFS	201	BioNTech SE	2020
NCT03815058 (phase 2)	Neoantigen mRNA	LNP	/	qw, q8w, up to 12 months	Advanced melanoma	Pembrolizumab	Intravenous injection	PFS	131	BioNTech SE	2019
NCT02035956 (phase 1/2)^114^	Neoantigen mRNA	LNP	/	0.13 mg,0.39 mg, W0, W2, W4, W8,	Multiple solid tumors		Intramuscular injection	ORR, AE	15	BioNTech RNA Pharmaceuticals GmbH	2018
NCT03639714 (phase 1/2)^232^	Neoantigen samRNA	Venezuelan equine encephalitis virus	/	ChAdV prime 1 × 10^12 vp SAM boosts 30–300 mg	NSCLC, gastroesophageal adenocarcinoma, urothelial carcinoma	Nivolumab Ipilimumab	Intramuscular injection	AE, ORR, RP2D	29	Gritstone bio, Inc.	2018
NCT03289962 (phase 1)	Neoantigen mRNA	LNP	/	q3w	Locally advanced or metastatic tumors	Atezolizumab	Intravenous injection	DLT, AE, RP2D	272	Genentech, Inc., BioNTech SE	2017
NCT03313778 (phase 1)	Neoantigen mRNA	LNP	/	40–1000 μg, 9 doses, q3w	Unresectable Solid Tumors	Pembrolizumab	Intramuscular injection	AE	108	ModernaTX, Inc., Merck Sharp & Dohme LLC	2017
NCT04534205 (phase 2)	HPV 16 E6 and E7 mRNA	LNP	/	Not mentioned	HPV16+ Head and Neck Cancer	Pembrolizumab	Intravenous injection	AE, OS, ORR	285	BioNTech SE	2021
NCT04382898 (phase 1/2)	KLK-2, KLK-3, PAP, HOXB13, and NKX3.1.	LNP	/	Not mentioned	Prostate cancer	Cemiplimab	Intravenous injection	DLT, ORR	115	BioNTech SE	2020
NCT03948763 (phase 1)	KRAS	LNP	/	9 doses, q3w	Metastasis NSCLC, Colorectal cancer and PDAC	Pembrolizumab	Intramuscular injection	DLT, AE	70	Merck Sharp & Dohme LLC	2019
NCT03164772 (phase 1/2)	MUC1, surviving, NY-ESO-1, 5T4, MAGE-C2, MAGE-C1	CVCM	/	80 μg, 12doses,	NSCLC	Durvalumab	Intradermal injection	AE	61	Ludwig Institute for Cancer Research	2017
NCT02410733 (phase 1)^22^	NY-ESO-1, MAGE-A3 and TPTE	LNP	/	7.2–400 μg, 7 doses, q3w	Advanced melanoma	PD-1 inhibitor	Intravenous injection	AE, ORR, DCR, DoR, PFS, OS	119	BioNTech SE	2015
NCT01302496 (phase 2)^233^	MAGE-A3, MAGE-C2, tyrosinase and gp100	autologous DC	CD70, CD40 ligand, TLR4	24 million DCs,4 doses, q3w	Advanced melanoma	Ipilimumab	Intradermal and intravenous injection	ORR	39	Bart Neyns	2011
**DNA vaccine**											
NCT03988283 (phase 1)	Neoantigen DNA	TDS-IM v2.0	/	6 doses, q3w followed with booster q3m	Recurrent brain tumor	/	Intramuscular injection+ electroporation	Safety and tolerability	10	Washington University School of Medicine	2023
NCT05743595 (phase 1)	Neoantigen DNA	TDS-IM	/	8 doses, q4w	Glioblastoma	Retifanlimab	Intramuscular injection+ electroporation	DLT	12	Washington University School of Medicine	2023
NCT04251117 (phase 1/2a)	Neoantigen DNA	Not mentioned	Plasmid Encoded IL-12	Not mentioned	Advanced Hepatocellular Carcinoma	Pembrolizumab	CELLECTRA®2000 EP Device	AE, immune response	36	Geneos Therapeutics	2020
NCT03122106 (phase 1)	Neoantigen DNA	Not mentioned	Mesothelin epitope	W1, W5, W9, W13, W17, W21	Resected pancreatic cancer	/	Intramuscular injection	AE	15	Washington University School of Medicine	2018
NCT03548467 (phase 1/2a)	Neoantigen DNA plasmid	injection DNA plasmid pUMVC4a vector	Bempegaldesleukin	3 mg, 14doses, q4w until week 50	Melanoma, NSCLC, RCC, UC, SCCHN	/	Intramuscular injection	AE, immune response, ORR, DoR, PFS	41	Nykode Therapeutics ASA	2018
NCT03199040 (phase 1)	Neoantigen DNA	TDS-IM	/	D1, 29, 57, 85 and D141	TNBC	Durvalumab	Intramuscular injection+ electroporation	AE, immune response	18	Washington University School of Medicine	2017
NCT05350501 (phase 2)	OncoMimics™ peptides, UCP2	Not mentioned	Montanide	Not mentioned	Colorectal cancer	Nivolumab	Not mentioned	ctDNA response	34	Enterome	2023
NCT05286060 (phase 2)	HPV16/18 E6/E7	Gx-17	Flt3L	2 mg, 1st day of week 1,2,4	HPV-positive head and neck cancer	Pembrolizumab	Intramuscular injection + electroporation	MPR	25	Yonsei University	2022
NCT05455658 (phase 2)	MDM2, YB1, SOX2, CDC25B, CD105 plasmid	Not mentioned	GM-CSF	3 doses qm + 1 dose q3m	Early stage TNBC	/	Intradermal injection	Immune response	33	University of Washington	2022
NCT03655756 (early phase 1)	Emm55 streptococcal antigen plasmid	Not mentioned	/	0.1 mg	Unresectable melanoma	/	Intratumoral injection	SAE, DLT	7	Morphogenesis, Inc.	2018
NCT02411786 (phase 1)	AR LBD	Not mentioned	GM-CSF	100 μg, 6 doses q2w followed with 4 doses q3m	Metastatic prostate cancer	/	Intradermal injection	AE, immune response	40	University of Wisconsin, Madison	2015
NCT02163057 (phase 1b/2)^234^	HPV16/18 E6/E7	Not mentioned	Plasmid Encoded IL-12	4 doses, q3w	HPV-Associated Head and Neck Cancer	Stand of care	Intramuscular injection + electroporation	AE	22	Inovio Pharmaceuticals	2014
NCT05269381 (phase 1)	Neoantigen peptides	Not mentioned	GM-CSF	D1,4,8,15, first cycle; d1 q3w	Advanced solid tumors	Pembrolizumab,	Intravenous injection	AE	36	Mayo Clinic	2022
NCT05010200 (phase 1)	Personalized multi-peptides	Not mentioned	Poly-ICLC, CDX-301	Not mentioned	Prostate cancer	/	Intracutaneous injection	AE	27	Ashutosh Kumar Tewari	2022
**Peptide vaccine**											
NCT03645148 (phase 1)^235^	Neoantigen peptides	Not mentioned	GM-CSF	100 μg, D1, D4, 8, 15, 22, 78 and 162	Advanced pancreatic cancer	Radiofrequency ablation	Subcutaneous injection	ORR, AE	7	Zhejiang Provincial People’s Hospital	2018
NCT03380871 (phase 1b)^175^	Neoantigen peptides	Not mentioned	Poly-ICLC	450 μg, W12D1, W12D4, W13, W14, W15, W19, W23	NSCLC	Chemotherapy and anti-PD-1	Subcutaneous injection	Safety and tolerability	38	BioNTech US Inc.	2017
NCT02956551 (phase 1)^136^	Neoantigen peptides	Autologous peptide	GM-CSF	W0, 1, 2, 4, 6, 8	Advanced lung cancer	/	Subcutaneous injection	AE, ORR	20	Sichuan University	2016
NCT02897765 (phase 1b)^97^	Neoantigen peptide	Not mentioned	Poly-ICLC	450 μg, W12D1, W12D4, W13, W14, W15, W19, W23	Melanoma, lung cancer and bladder cancer	Nivolumab	Subcutaneous injection	Safety and tolerability	34	BioNTech US Inc.	2016
NCT05937295 (phase 1)	DNAJB1-PRKACA fusion transcript-based peptid	Montanide ISA 51 VG	XS15	2 doses, q4w followed with 1 dose after 1 year	Fibrolamellar hepatocellular carcinoma	Atezolizumab	Subcutaneous injection	Immunogenicity and AE	20	University Hospital Tuebingen	2023
NCT05283109 (phase 1b)	P30-linked EphA2, CMV pp65, and survivin peptides	Not mentioned	Poly-ICLC	200–400 μg, 5 doses priming, d1-d22 followed with 2 doses booster d84, d140	Glioblastoma	Radiation therapy and temozolomide	Not mentioned	DLT	36	Duke University	2023
NCT05609994 (phase 1)	IDH1 peptide	Not mentioned	/	12 doses, d1, d15, d1 q4w	Recurrent IDH1 mutant lower grade glioma	Vorasidenib	Intracutaneous injection	Safety and efficacy	48	Duke University	2023
NCT04206254 (phase 2/3)	Heat shock protein gp96-peptide	Not mentioned	/	25 μg,8 doses, qw	Liver cancer	/	Subcutaneous injection	RFS	80	Cure&Sure Biotech Co., LTD	2019
NCT03879694 (phase 1)	Survivin long peptide	Not mentioned	GM-CSF, IFA	4 doses, q2w followed with q3m up to a year	Metastatic neuroendocrine tumor	Octreotide Acetate	Subcutaneous injection	AE	14	Roswell Park Cancer Institute	2019
NCT02960230 (phase 1)^236^	H3.3.K27M epitope peptide	Montanide® ISA-51 VG	poly-ICLC	8 doses, q3w	diffuse midline glioma	Nivolumab	Subcutaneous injection	AE, OS	50	University of California	2016
NCT02454634 (phase 1)^237^	Mutated IDH1 peptide	Not mentioned	imiquimod	300 μg, 8 doses, q2w-q4w	Grade III-IV gliomas	/	Subcutaneous injection	RLT, immune response	39	National Center for Tumor Diseases, Heidelberg	2015
NCT01706458 (phase 2)	PAP peptide	autologous DC	GM-CSF	W0, W2, W4	Metastatic prostate cancer	pTVG-HP plasmid DNA vaccine	Intravenous injection	Immune response	18	University of Wisconsin	2013
NCT01846143 (phase 1)^227^	pIRS2 and BRCA3 peptide	Montanide ISA-51 VG	incomplete Freund’s adjuvant and Poly-ICLC	100 mcg and 200 mcg, 8 doses, D1, 8, 15, 36, 57, 78	High-risk melanoma	/	Subcutaneous and intradermal injection	AE	15	University of Virginia	2013
NCT01461148 (phase 1/2a)^238^	AIM2, HT001, TAF1B neoantigen	Montanide® ISA-51 VG	/	100 μg, qw × 4 every 3 month	MMR-deficient colorectal cancer	/	Subcutaneous injection	Immune response, ORR, AE	22	Oryx GmbH & Co. KG	2011

*CT* Clinical trial, *mRNA*, Message RNA, *LNP* Lipid nanoparticle, *RFS* Recurrence free survival, *DC* Dendritic cell, *DLT* Dose limiting toxicity, *AE* Adverse events, *DFS* Disease free survival, *PFS* Progress free survival, *ORR* Overall response rate, *samRNA* Self-amplifying RNA, *RP2D* Recommended phase 2 dose, *OS* Overall survival, *KLK* Kallikrein, *PAP* Prostatic acid phosphatase, *HOXB4* Homeobox gene B4, *KARS* Kirsten rat sarcoma virus, *NSCLC* Non-small cell lung cancer, *PDAC* Pancreatic ductal adenocarcinoma, *MUC1* Mucin 1, *NY-ESO-1* New York esophageal squamous cell carcinoma-1, *MAGE* Melanoma-associated antigen, *TPTE* Transmembrane phosphatase with tensin, *DCR* Disease control rate, *DoR* Duration of response, *TLR* Toll like receptor, *ctDNA* Circulating tumor DNA, *RCC* Renal cell carcinoma, *UC* Urothelial cancer, *SCCHN* Squamous cell carcinoma of the head and neck, *TNBC* Triple negative breast cancer, *HPV* Human papillomavirus, *UCP2* Uncoupling protein 2, *Flt3L* Fms-related tyrosine kinase 3 ligand, *MPR* Major pathological remissions, *MDM2* Murine double minute 2, *YB1* Y-box binding protein 1, *SOX2* Sex-determining region Y-box 2*, CDC25B* Cell division cycle protein 25B, *SAE* Serious adverse event, *AR LBD* Androgen receptor ligand-binding domain, *GM-CSF* Granulocyte-macrophage colony-stimulating factor, *Poly-ICLC* Polyriboinosinic-polyribocytidylic acid-poly-L-lysine carboxymethylcellulose, *IDH1* Isocitrate dehydrogenase 1, *IFA* Incomplete Freund’s adjuvant, *RLT* Regime limiting toxicity, *BRCA* Breast cancer susceptibility gene, *AIM2* Absent in melanoma 2, *TAF1B* TATA box binding protein associated factor B, *MMR* Mismatch repair

## Combined therapy

**Fig. 6:**
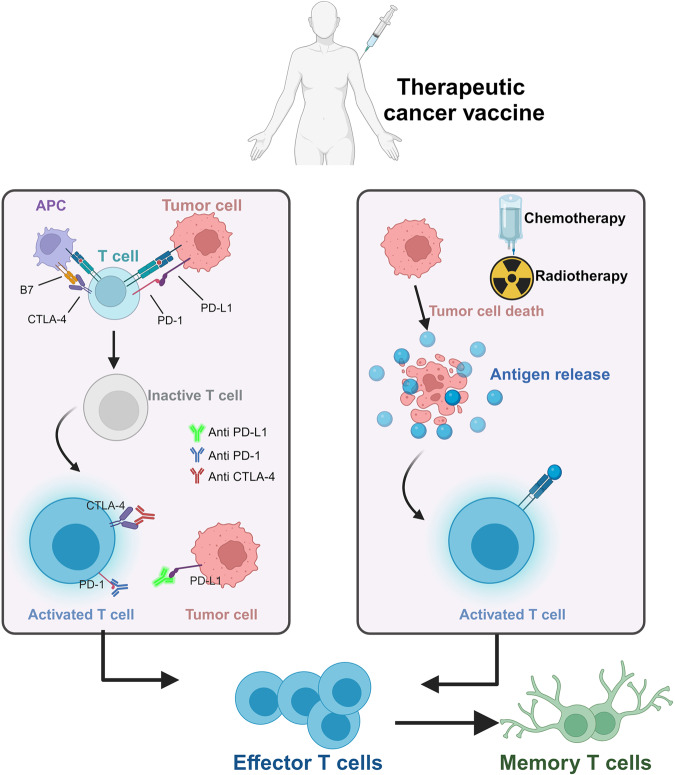
Mechanistic diagram of the different combination therapies after the immunization of cancer vaccine in patient. ICIs unblock immunosuppression and stimulate T cell activation by blocking signaling pathways on the surface of T cells and cancer cells. Conventional therapies, such as chemotherapy and radiotherapy, complement this process by directly affecting tumor cells or indirectly by eliminating immune suppressor cells. Different therapy combinations can activate effector T-cells, enhancing the clinical efficacy of neoantigen cancer vaccines. ICIs Immune checkpoint inhibitors, APC Antigen presentation cell, PD-1 Programmed cell death protein 1, PD-L1 Programmed cell death-ligand 1, CTLA4 Cytotoxic T-lymphocyte-associated antigen 4

Specific combined strategies have been devised to address diverse treatment challenges considering the distinctive pathological characteristics of each tumor, different drug resistance mechanisms, and the advantages and disadvantages of distinct vaccine platforms. Patients with advanced cancer, particularly those with multiline therapy, frequently encounter a high tumor burden, multiple drug resistance, and immune deficiency. Achieving disease remission by simultaneously reducing the tumor load and enhancing the body’s anti-tumor immunity has been the primary aim in these patients (Fig. 6). Consequently, several combination strategies have been implemented to induce tumor remission by minimizing the tumor burden and boosting anti-tumor immunity. The initial step involves surgery, chemotherapy, or radiotherapy to reduce or eliminate the tumor cells. The subsequent stage focuses on establishing immunological memory to prevent tumor recurrence and stimulate specific immunity to eradicate any remaining tumor cells through vaccination. This comprehensive approach can improve clinical outcomes and reduce the risk of disease progression.

### Chemotherapy

Chemotherapy can directly affect tumor cells and enhance therapeutic efficiency through immune modulation. Marij et al. demonstrated that an SLP therapeutic cancer vaccine plus carboplatin and paclitaxel improved survival rates in mice.^172^ This improvement was attributed to chemotherapy-induced changes in bone marrow cell population composition. Subsequently, these finding were further validated in clinical trial (NCT02128126), where the combination of the HPV16 SLP vaccine ISA101 with chemotherapy not only depleted the abnormal myeloid cells, but also lead to increased T cell proliferation in response to recall antigens and resulted in a strong and sustained antigen-specific T cell response in HPV16-positive advanced stage cervical cancer patients, which was associated with the clinical benefit.^172,173^ These findings demonstrate that chemotherapy can modulate immune responses and enhance cancer vaccine efficacy.

### Immune checkpoint inhibitor

Recently, ICIs have shown promise as cancer treatment approaches. However, the effectiveness of ICI therapy remains limited since only a few patients benefit from it owing to inadequate pre-existing cytotoxic T cell response.^174^ Cancer vaccines can active T cells to transform the tumor immune microenvironment (“cold” to “hot” tumor). Combining ICIs with cancer vaccines presents an enticing strategy for enhancing the immune therapy response, warranting further exploration in future clinical studies. A personalized SLP cancer vaccine, NEO-PV-01, led to major pathological responses in 9 patients with melanoma with limited or only partial responses to nivolumab. Moreover, NEO-PV-01 plus nivolumab induced epitope spread, leading to the release of neoantigens that provided new targets for T cells.^97^ Further clinical investigations may establish NEO-PV-01 plus chemotherapy and anti-PD-1 as a potential first-line treatment for non-squamous NSCLC.^175^ Despite the immunosuppression caused by pemetrexed plus carboplatin, all trial patients generated a robust immune response and the MHC II expression in the TME post-vaccination, thereby developing a strong CD4^+^ T cell response. Epitope spread toward the most common driver mutations KRAS G12C and G12V was found in 7 out of 19 patients.^175^ In another phase 1b trial (NCT03289962), the efficacy of an RNA-lipoplex neoantigen-based vaccine named BNT122 combined with atezolizumab was assessed in patients with locally advanced or metastatic solid tumors. Among the 144 patients enrolled in the trial, 73% exhibited an ex vivo immune response after receiving the combination therapy. The vaccine-specific T cells were detected at frequencies exceeding 5% in peripheral blood and displayed a T effector memory phenotype with high PD-1 expression levels. The majority of adverse events reported were of grade 1/2 and no dose-limiting toxicities (DLT) were observed. Tumor assessment in 108 patients revealed a disease control rate of 57%, with 1 patient achieving a complete response and 8 patients experiencing partial remission.^115^ However, it’s worth noting that initial clinical trials involving patients with advanced cancers treated with personalized neoantigen cancer vaccines in combination with ICIs did not demonstrate superior ORRs compared to ICIs alone.^176^ These daunting clinical results were likely related to the late tumor stage and heavy pretreatment. Therefore, further studies should identify candidate patients and appropriate tumor stages for this combination therapy.

The aforementioned clinical trials are limited by their single-arm design, making it difficult to solely attribute the observed cytotoxic T cells, epitope spread, and clinical efficacy to the cancer vaccines. Randomized trials comparing cancer vaccine combined with ICIs to ICIs alone are crucial to pinpoint the functional relevance of the cancer vaccine. Recent groundbreaking information highlights the success of a phase 2b clinical trial conducted by Moderna, involving their personalized cancer vaccine mRNA-4157 combined with pembrolizumab in patients with resected stage III/IV melanoma. Notably, the combination strategy achieved the primary endpoint, leading to a significantly reduced risk of recurrence and death (hazard ratio=0.56 [95% CI, 0.31–1.08], *p* = 0.0266).^98^ Moreover, several randomized controlled clinical trials are currently underway to investigate the concurrent utilization of cancer vaccines and ICIs as a first-line treatment. The objective of these trials is to ascertain whether the synergistic integration of cancer vaccines and ICIs can augment the anti-tumor effectiveness of cancer vaccines (NCT03815058 and NCT03897881).

## Dosage and the route of administration

Understanding the driving forces behind T cell activation, subgroup differentiation and long-term memory development are crucial to comprehensively understand cancer vaccine applications. Maximizing the immune response and antitumor efficacy of vaccination requires careful evaluation of the optimal dosing, administration route, and frequency in preclinical and clinical trials.

### Optimal drug concentrations

Determining the appropriate dose is of utmost importance since the vaccine at a low dose may render the antigen ineffective, whereas a higher dose could result in DLT, cytokine-releasing syndrome, or a strong innate immune response.^127^ Previous clinical trials have demonstrated that a higher vaccination dose within the safety range improved immune response and anti-tumor efficacy.^22,177^ The antigen dose was synchronized with the initial cytotoxic T cell response and the subsequent generation of memory T cell.^178^ Immunized mice exposed to high antigen concentrations produced T cells with lower functional effector capabilities, while those stimulated with very low antigen concentrations exhibited significantly higher functional avidity.^179^

### The route of administration

Besides the dose, the delivery route is a critical variable to consider. Each delivery route has its set of challenges that influence the best delivery method to overcome (Table 2).^180^ For example, intradermal injection facilitates efficient antigen delivery by the Langerhans cells and mesenchymal DCs in the epidermis and dermis, respectively.^181^ Skeletal muscle only contains a few immune cells; therefore, conventional vaccines frequently contain adjuvants that can arouse an inflammatory response at the injection site to recruit APCs to induce an anti-tumor response by intramuscular injection.^182^ The intravenous administration of cancer vaccines faces difficulties, such as rapid drug degradation and serum protein aggregation.^183^ The stability and uptake ratio of nucleic acid vaccine can be improved by carrier packaging. Biodistribution after intravenous administration is also crucial; for example, cationic LNP-mRNA vaccines primarily target the liver after systemic administration, providing insights into liver-targeted therapies.^184^ Different vaccination methods may result in varied antigen accumulation and immune responses at distinct sites in the body. For instance, vaccines with human serum albumin as an adjuvant showed stronger Th2 effects following oral primary immunization in mouse models compared to immunization via intramuscular or subcutaneous routes.^185^ A DNA vaccine encoding key rheumatoid arthritis autoantigens administered intramuscularly reduced the disease incidence and severity better than subcutaneously and intravenously, without local accumulation.^186^ Direct injecting adjuvants, tumor-lysing viruses, immunostimulant-carrying mRNAs, and activated autologous or allogeneic DCs to the tumor site can lead to immediate TME alterations without requiring antigen prediction.^187,188^ However, this approach is suitable for only individuals with treatable conditions.^189^

Additionally, antigen expression and delivery can be limited by lymph node drainage. Multiple-site injections have shown promise in addressing this issue. For instance, multiple-site injections of rabies vaccines increased virus-neutralizing antibody titers in the human body.^190^ Similarly, distributing a cancer vaccination dose over several lymphatic drainage areas could enhance antigen-specific T cell responses.^191^ This simple and cost-effective strategy holds the potential to improve the effectiveness of cancer vaccines.

Furthermore, insights into antigen kinetics can provide valuable information for further improvements in clinical trials, and mouse models can also be instrumental. By administering different vaccination schedules at a fixed cumulative antigen dose, researchers can determine the most effective antigenic stimulation of CD8^+^ T cells. Research has shown that antigenic stimulation that increased exponentially over days stimulated antiviral immunity more effectively than a single or multiple doses with daily equal dosages.^192^ The administration timing, total dose, and prime and boost intervals were thoroughly studied to justify the initial clinical program.^21^ However, because of insufficient large-scale clinical trials for cancer vaccines and the high degree of heterogeneity among patients with cancers, obtaining accurate and stable dose-response relationships remains challenging. Therefore, optimal experimental models should be explored to clarify the ideal dosage and delivery method for each cancer vaccine to provide the maximum clinical benefit for patients.

## Clinical challenges

Although an increase of anti-tumor effector cells have been detected after vaccination, only modest clinical benefits have been achieved in small-scale populations. Which variables restrict cancer vaccine efficacy? What difficulties do the existing clinical applications encounter? The following section will focus on the current clinical challenges associated with cancer vaccines.

### Animal models and preclinical innovations

It is a great challenge to study the interactions between immune cells and tumors as well as to assess the alterations in immune cell phenotype and function after anti-tumor therapies due to the intricacies of the human immune system and the complexity of the TME. While mouse models are invaluable for gaining fundamental insights into basic biological processes, they have limitations when it comes to investigating human tumor biology, the intricate TME, and the mechanisms underlying resistance to immunological therapy. These limitations underscore the importance of combining insights from mouse models with clinical research to develop a comprehensive understanding of cancer and its treatment.^193^

Humanized mice have begun to fulfill this gap by generating mice with Prkdc^scid^ mutation, Rag1/Rag2 deficiency or disruption of the IL2rg locus disruption to reduce host innate and adaptive immunity.^194^ These severely immunodeficient mice support the growth of cell line-derived xenografts (CDX) and patient-derived xenografts (PDX) after reconstruction of the human immune system. Several key aspects of humanized mice restrict their application in cancer vaccine research, including the underdevelopment of mature humanized host immune cell populations (particularly T cells and plasmacytoid DCs for cancer vaccines), insufficient HLA molecules in standard immune-deficient mouse strains, and absence of well-developed lymph node structures and germinal centers, leading to an inability to generate antigen-specific cellular immunity.^195^ In addition, the accurate prediction of neoantigens relies on comparative calculations of tumor tissue and PBMC sequencing data from the same donor, which this dual humanized strategy depends on the availability of cancer patients willing to provide blood and tumor samples for preclinical studies. Currently, commonly used mouse models for the immune system humanization include the human peripheral blood mononuclear cell (huPBMC) humanized mice model and the human hematopoietic stem cell (huHSC) humanized mice model. huPBMC humanized mice model can rapidly reconstitute mature and activated human T-lymphocytes, but cannot successfully reconstitute the myeloid system including DCs, or present antigens.^194^ The anti-tumor effect of carbonic anhydrase 9 antibody was tested using allogeneic huPBMC in NOD/SCID/IL2Rγ (-/-) mice by transplanting a novel orthotopic renal cell carcinoma CDX and found that the antibody of this tumor-specific antigen inhibited tumor growth.^196^ Transplanted human PBMC mediates acute graft-versus-host disease and body weight loss. In severe cases, death owing to graft-versus-host disease significantly impacts drug efficacy experiments by shortening the vaccination time window.^197^ The huHSC humanized mice model is able to rebuild various human immune cells, such as T, B, and myeloid cells, however, the incomplete development of mature human innate cell lineages, coupled with the HLA expression, imposes limitations on the use of huHSC models in cancer vaccine studies.^198^ In many studies, the introduction of transgenic expression of HLA molecules has led to improved development and survival of human T cells, making these models more suitable for investigating cancer vaccines.^199^ An example of this strategy was the transduction of a lentiviral vector containing HLA-A*0201 and A*2402-restricted TCR specific for the WT1 antigen into CD34^+^ hematopoietic stem cells to express HLA-A24. With this approach, WT1-specific CD8^+^ T cells were detected in both thymus and peripheral tissues of these mice.^200^ Based on the precise sequencing of CDX or PDX, rapid construction of humanized mice using matched-HLA human PBMCs or hematopoietic stem cells allows for a more comprehensive immune system through strategies, such as thymus transplantation, which is still important for assessing the immunogenicity and tumor-suppressing efficiency of future neoantigen cancer vaccines.

The application of new technologies has led to many innovations in preclinical research on cancer vaccines with technological advancements. For example, high-throughput technologies can accelerate the discovery of neoantigens, and technologies, such as artificial intelligence and machine learning, can accelerate data analysis and simulation prediction to assist vaccine development decision-making process (described in detail in the section “neoantigen cancer vaccine”). Gene editing technologies can also optimize vaccine design and screen vaccine targets. For example, researchers found that tumor cells sensitive to interferon-β were transformed into drug-resistant tumor cells by knocking out the interferon-β-specific receptor through CRISPR-Cas9 technology, which subsequently caused them to release the immunomodulatory factors interferon-β and GM-CSF.^201^ These modified therapeutic tumor cells can effectively activate signaling pathways specific to antigen-triggered T cell activation and stimulate the trafficking of immune cells, which may be beneficial for the further application of cancer vaccines.^201^

Developing an appropriate in vitro assessment model to characterize the function of antigen-specific T cells is critical to therapeutic cancer vaccine research. Two promising potential models are PDX in highly immunodeficient mice and patient-derived organoids.^148,202^ PDX in highly immunodeficient mice offers a valuable platform for evaluating the anti-tumor efficacy of antigen-specific T cells. This model preserves the endogenous expression, natural processing, and presentation of neoepitopes, making it a reliable representation of the human tumor environment.^203^ In contrast, 3D patient-derived organoids represent another innovative preclinical therapeutic model, involving a tumor organoid-T cell co-culture system, that maintains tumor heterogeneity and the TME necessary for neoantigen presentation and T cell recognition. Using this model, researchers can precisely measure each patient’s susceptibility to immunotherapy, making it an excellent tool for personalized cancer treatment.^203,204^ Researchers can gain critical insights into neoantigen-reactive T cells functionality and effectiveness by utilizing these in vitro assessment models, thereby facilitating the development of precise and individual immunotherapies for patients with cancer.

### Overcome the suppression of tumor immune microenvironment

**Fig. 7:**
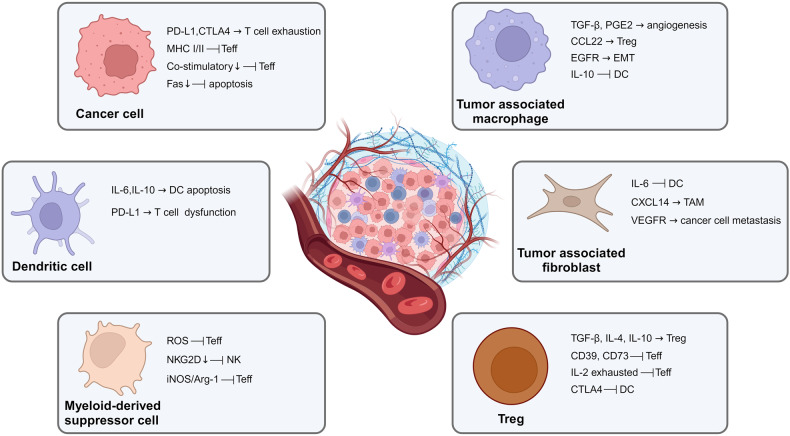
Roles of cancer cells and immunosuppressive cells in tumor immune microenvironment. Immunosuppressive cells in the TME, such as Tregs, M2 macrophages, myeloid-derived suppressor cells, PD-L1-positive DCs, cancer-associated fibroblasts, and tumor cells form a suppressive ecological niche at the tumor site through the expression of immunosuppressive signals. TME Tumor microenvironment, Tregs Regulatory T cells, PD-L1 Programmed cell death-ligand 1, DC Dendritic cell, CTLA4 Cytotoxic T-lymphocyte-associated antigen 4, MHC Major histocompatibility complex, Teff, Effector T cell, IL Interleukin, ROS Reactive oxygen species, NK Natural killer cells, iNOS Inducible nitric oxide, Arg-1 Arginase 1, TGF-β Transforming growth factor-β, PEG2 Prostaglandinum E-2, CCL22, C-C motif chemokine ligand 22, EGFR Endothelial growth factor receptors, EMT Epithelial-mesenchymal transition, CXCL14 CXC-chemokine ligand 14, TAM Tumor-associated macrophage, VEGFR Vascular endothelial growth factor receptors

The tumor microenvironment is a complex system that encompasses a variety of immune components, including both innate and adaptive immune cells, extracellular immune factors, and cell surface molecules.^205^ However, the presence of immunosuppressive cells, such as regulatory T cells (Tregs), M2 macrophages, myeloid-derived suppressor cells (MDSC), programmed cell death-ligand 1 (PD-L1)-positive DCs, and cancer-associated fibroblasts creates an inhibitory niche within the TME that protects the tumor from immune attack.^206^ These cells in the TME release abundant immunosuppressive signals, including PD-L1, transforming growth factor-beta (TGF-β), and vascular endothelial growth factor, into the microenvironment and act directly or indirectly on effector T cells to suppress tumor immunity.^207^ For instance, Tregs inhibit the activation and proliferation of effector T cells, whereas M2 macrophages promote tumor growth by inducing angiogenesis and tissue remodeling. PD-L1-positive DCs suppress T cell function by presenting antigens to them, and cancer-associated fibroblasts secrete extracellular matrix components that physically restrict T cell infiltration into the tumor.^207^ Figure 7 summarizes the roles of the different components in creating an immunosuppressive microenvironment.

A greater understanding of the TME complexity and diversity and its impact on treatment response is being revealed as research progresses. Therefore, strategies from the following four perspectives may effectively reverse the suppressive TME toward improving the vaccine’s anti-tumor efficacy: (1) immunosuppressive cell depletion, (2) immune checkpoint inhibition, (3) targeting the tumor structure, and (4) enhancing T cell activation or survival signaling.

Tumors often promote immunosuppressive cell subpopulation differentiation or activation to evade immune attacks. Targeting and eliminating these immunosuppressive cells can improve tumor vaccine efficacy. For example, low continuous doses of cyclophosphamide selectively target and eliminate Tregs while promoting Th17 differentiation, thereby supporting host anti-tumor immunity.^208,209^ The stand of care chemotherapy with carboplatin/paclitaxel can deplete HPV-specific Treg and MDSCs, which may enhance the efficacy of HPV16 SLP cancer vaccine.^173^ Recent approaches have exploited macrophage plasticity by reprogramming M1 macrophages to an M2 anti-tumor phenotype, through targeting molecules, including PI3K, STAT3 and IDO-1.^210–212^ Developing immunotherapy-based combination therapies (e.g., CTLA4, PD-1/PD-L1) has emerged as a prominent trend in the field of cancer vaccine application (discussed in the combined therapy section above). Antigen-specific T cells generated by cancer vaccines often express suppressor molecules, and the PD-1/PD-L1 axis is involved in signaling mediated by the TCR recognition of antigens, providing a mechanism through which tumor cells can resist attacks by T cells.^213,214^ Therefore, blocking the PD-1/PD-L1 signaling pathway can release the immune system “brake”, reversing the immunosuppressive microenvironment. The TME contains a plethora of immunosuppressive cytokines, mainly TGF-β and IL-10. Inhibiting TGF-β signaling in T cells has been demonstrated to enhance their capacity to infiltrate tumor tissue, proliferate, and exert anti-tumor responses in prostate cancer models.^212^ Another strategy involves the intratumoral delivery of immunomodulatory cytokines, such as IL-2, IL-12, and GM-CSF, to promote anti-tumor immune responses.^215–217^ However, maintaining the correct balance of cytokines within the TME to prevent inducing harmful inflammatory responses is challenging. Cancer cells manipulate the TME to support their growth and survival, which includes stimulating angiogenesis for continuous access to oxygen and nutrients. Additionally, inhibiting angiogenesis using antibodies targeting vascular endothelial growth factor or multi-tyrosine kinase receptor inhibitors can inhibit neoangiogenesis and induce the tumor vasculature normalization, activating the tumor-infiltrating lymphocytes’ infiltration into the TME.^218^ Numerous studies have revealed that modulating the T cell signaling pathway can help protect T lymphocytes from the immunosuppressive effects of TME. For example, inhibiting cholesterol esterification in T cells by targeting ACAT1 enhanced their effector function and proliferation, whereas mRNA-encoded constitutively active STING^V155M^ maximized CD8^+^ T cell responses.^219,220^

In summary, effector immune cells must overcome multiple suppressive networks and activation barriers within TME. Targeted inhibition of pivotal factors within these networks can establish a favorable environment for anti-tumor immune responses, ultimately improving cancer vaccines efficacy.

### Identify the optimal candidates and expand the therapeutic range of cancer vaccines

Immune evasion is a prominent characteristic of cancer, and therapies aimed at restoring immune surveillance have shown significant effectiveness especially in cancers with a high tumor mutation burden (TMB). In addition, the higher the TMB, theoretically, the more non-synonymous tumor cell mutations that can be detected and the more neoantigens that may contain immunogenic properties bound to MHC.^205^ “High-quality” neoantigens crucially elicit a strong and sustained T cell immune response. Identifying suitable patients for neoantigen cancer vaccine application involves accurate HLA typing, evaluating antigens serological activity, and assessing the patient’s neoantigen merits.^9^ Consequently, tumors with high TMB, including melanoma, NSCLC, and bladder cancer, have been the initial focus of neoantigen-based cancer vaccine research, and early data have shown promising efficacy.^40,97,221^ However, potential immunogenic neoantigens require further exploration to broaden cancer vaccine application for tumors with low TMB, including glioblastoma and microsatellite stable colorectal cancer (MSS CRC).^130^ Studies have found that all tumors in patients with MSS CRC contain HLA-I-type clonal neoantigens; however, these were expressed at low levels. The low expression of these neoantigens contributes to poor T cell infiltration and ICI responses.^222^ Since insufficient antigenic stimulation may cause early immune escape from cancer, therapeutic vaccination can be employed using therapeutic priming, including anti-CD40 and ICIs.^222^ With the breakthroughs in immunotherapy, particularly the success of ICIs in inflamed tumors, the scope of immunotherapy is expanding to encompass immune-excluded and immune-desert tumors. Even within tumors with low passenger mutation rates, advanced high-throughput techniques have enabled identifying immunogenic neoepitopes detectable by CD8^+^ T cells. For example, despite the low mutation rate in PDAC, pathogenic germline variants were identified using the MSK-IMPACT panel, and personalized mRNA cancer vaccines were established to induce individual immune response against neoantigens specific to this tumor.^116,223^ Research has demonstrated that cancer vaccines can activate cytotoxic T cells in non-inflammatory tumors displaying immune exclusion or desert phenotypes, achieved through the integration of integrates next-generation sequencing and mass spectroscopy data.

Cancer vaccines are under development in the pipelines primarily in patients with advanced tumors. Patients who have undergone one or several prior regimens may encounter challenges in responding to cancer vaccines because of severe damage to bone marrow function and immune system suppression caused by multiple drug therapies.^97,224^ To address these issues and provide treatment access to more patients with advanced disease, there is a need to expand the use of cancer vaccines from later treatment lines to first-line treatment. Therefore, the risk of patients becoming severely ill to receive treatment can be reduced.^175^ However, cancer vaccine efficacy in advanced diseases is limited because of disease progression and these patients’ poor physical condition. Another important consideration is the personalized cancer vaccine manufacturing time, which typically takes at least 2 months. This duration renders these vaccines unaffordable for patients with advanced stage cancer. One approach to tackle this challenge is to focus on early-stage diseases where patients have not yet established immune tolerance. Utilizing neoadjuvant therapy in patients with early-stage cancer and employing cancer vaccines in post-operative therapy may extend the therapeutic time window and maximize individual neoantigen cancer vaccine efficacy under conditions of low tumor load and better physical scores. Therefore, cancer vaccine-related studies are more desirable in the early stages of the disease or postoperative patients, including HPV-related cervical intraepithelial neoplasia and high-risk non-muscle-invasive bladder cancer.^225,226^ Recent advances in cancer vaccines have been promising. For instance, BioNTech reported preliminary clinical data from a phase 1 trial of an individual mRNA-based neoantigen-specific immunotherapy combined with atezolizumab and mFOLFIRINOX chemotherapy in patients with resected PDAC. This approach generated cytotoxic and durable neoantigen-specific T cells, potentially delaying recurrence in patients with PDCA.^116^ Similarly, Moderna/Merck’s personalized cancer vaccine mRNA-4157 in combination with pembrolizumab as an adjuvant treatment for patients with resected stage III/IV melanoma significantly reduced the risk of recurrence and death, providing further evidence regarding the potential for the future commercialization of personalized neoantigen cancer vaccines in postoperative patients.^98^

### Evaluation of clinical outcomes

Currently, there is only one approved cancer vaccine on the market, while all other cancer vaccine-related products remain under pre-marketing clinical studies. The success of these clinical trials hinges on the careful design of trial endpoints. As shown in Table 2, ensuring safety is paramount, necessitating diligent monitoring and evaluation of safety indicators, including adverse events and side effects, to safeguard the patient’s well-being. Designing clinical trials with a greater focus on the clinical benefits to patients is crucial since prolonged patient survival is the most satisfactory and trustworthy outcome of a cancer vaccine efficacy assessment. Although initial small-scale clinical trials have shown modest improvements in cancer vaccine efficacy among patients with tumors, most lacked the rigor of a two-arm randomized controlled trial. Consequently, evaluating the clinical benefits of cancer vaccines for patients with tumors is crucial through large-scale phase 3 randomized controlled clinical trials. These trials can adopt efficacy indicators including PFS, OS, and RFS as the gold standard for evaluating vaccine efficacy. A recent clinical trial design led by Merck and Moderna, involved a phase 3, randomized, double-blind study that evaluated the combination of mRNA-4157 and pembrolizumab versus the efficacy of pembrolizumab alone in 1089 patients with melanoma (NCT05933577). In this trial, RFS was the primary endpoint, providing valuable insight into the product’s efficacy and validating its potential for melanoma treatment.

The fundamental objective of cancer vaccines is to activate the immune system against tumor cells. Several immune metrics are considered to evaluate the immunological efficacy of cancer vaccine, and assessing antigen immunogenicity after vaccination has become the important endpoints in clinical trials. Interferon-γ Enzyme-Linked Immuno-Spot (ELISpot) could identify T cell clones that respond to the specific antigen epitopes, while flow cytometry analysis of peptide-MHC conjugates (e.g., tetramer) enables detecting the number of neoantigen-specific T cells in PBMCs.^97,116^ Researchers have previously used intracellular cytokine staining (ICS) to determine activation status and cytotoxicity of T cells after in vitro stimulation with matched epitopes or tumor lysates.^67,227^ TCR sequencing of tumor-infiltrating lymphocytes and peripheral blood T cells can be informative, particularly regarding T cell phenotypes and antigen-specific interactions. Bulk TCR sequencing is a valuable tool that offers insight into the diversity and clonality of the TCR repertoire.^228^ Therefore, this technique provides an overall view of T cell populations that respond to tumor neoantigen vaccines when many T cells are analyzed together. Additionally, single-cell TCR sequencing analysis is a more focused approach that provides a precise and detailed characterization of individual T cell responses.^229^ Another study identified antigen-specific clonal expansion by TCR sequencing of T cells cultured for a short period after peptide stimulation and combined it with a bioinformatics platform (MANAFEST).^230^ This cutting-edge method reveals dynamic changes at the clonotypic level in cancer neoantigen vaccine-specific T cells. This demonstrates the high specificity and efficacy of T cell immune responses triggered by tumor neoantigen vaccines at the cellular level. Moreover, studies have shown that follow-up after neoantigen vaccination in patients with hepatocellular carcinoma and monitoring of individualized neoantigen mutations in peripheral blood circulating tumor DNA could facilitate real-time evaluation of the clinical response.^228^ Longitudinal analysis of blood samples can also provide relevant information on changes in the immune system dynamics over time.

Epitope spreading refers to releasing additional epitopes after cancer cell destruction using cancer vaccines, thereby diversifying the T cell repertoire and eliciting a broader T cell immune response. A previous study demonstrated that individual neoantigen cancer vaccines can elicit responses against KRAS G12C and G12V mutations, and long PFS is associated with epitope spreading.^97,175^ Thus, epitope spreading has become an indispensable marker of vaccine effectiveness. Additionally, a re-biopsy analysis of the tumor tissue post-vaccine treatment can be conducted to provide evidence of vaccine effectiveness. The efficacy assessment of the major pathological responses can be performed by analyzing the residual tumor content of the tissue core biopsies pre-and post-treatment. The infiltration of CD3^+^ T cells at the tumor site post-treatment was associated with a good clinical response.^97^ In the future, novel technologies, such as multiplex immunofluorescence, slide-DNA-seq, and TARGET-seq, will enable a more refined characterization of tumor tissue changes following vaccination. These advancements will provide dynamic and precise predictions of disease outcomes and immunotherapy responses.

By combining comprehensive clinical and immunological assessments, researchers can gain a deeper understanding of the effects of cancer vaccines on patient outcomes and immune system activation. This data-driven approach not only paves the way for potential regulatory approvals but also opens doors to advancements in cancer treatment. Furthermore, with solid evidence from well-designed clinical trials, cancer vaccines have the potential to revolutionize cancer treatment and improve patient outcomes, providing new hope for the fighting cancer.

### Accelerate vaccine manufacturing

Cancer vaccine technologies are still in the early stages of development, and various formats have been explored in preclinical and clinical studies for off-the-shelf and personalized cancer vaccines. Optimal delivery conditions, including the choice of adjuvant and schedule of administration, should be established for all forms of personalized neoantigen vaccines. The vaccine design will significantly influence the strength of the immune response to candidate neoantigens.

The manufacture of cancer vaccines is a significant challenge. Shared antigen cancer vaccines can be mass-produced as ready-to-use, reducing production times and costs. Since each personalized neoantigen vaccine is treated as a stand-alone drug, demanding for rapid drug production, scalability, and cost control is needed to enable rapid clinical application. This differs from the conventional pharmaceutical development approach, focusing on bulk upscaling. Therefore, adopting a synthetic vaccine technology that facilitates fast and cost-effective production through a simple, robust, invariant, and good manufacturing practice (GMP)-compliant process is essential to address these complexities. Favorable solutions may involve completely digitizing the production processes, platforming the production quality control, modularizing production plants, and intelligentizing production hardware.^231^ There is a need for better coordination of every aspect of production and preparation, automated management tracking of every step, and acceleration of the entire vaccine preparation. These advancements have enabled the creation of scaled parallelized miniaturized production lines and significantly enhanced the overall efficiency and accessibility of cancer vaccines for clinical applications.

## Conclusion and perspectives

Over the past few decades, a substantially enhanced understanding of how cancer cells elude the immune system detection led to remarkable progress in cancer immunotherapy. Immune checkpoint inhibitors and adoptive cell therapies have demonstrated tumor regression-inducing ability inpatient with hematological and solid tumor cohorts. These advances have showcased the viability of tumor immunotherapy, and cancer vaccine development has entered a phase of rapid growth by mimicking natural immunity. Therapeutic cancer vaccines represent an innovative trajectory for future immunotherapy, primarily because of their safety profile, specificity, and ability to confer enduring immune memory. Cancer vaccine research is undergoing an intense surge in early-stage clinical investigations globally. Predefined antigen cancer vaccines targeting TAA have been investigated in prior research, although their efficacy is limited. Personalized cancer vaccines targeting neoantigens have garnered significant interest. However, their widespread application remains constrained by challenges, such as the intricacy of neoantigen identification, complexities of rapid manufacturing, and intricacies of detecting clinically meaningful immune responses. Less-successful clinical trials have gained valuable insights, guiding the necessary adjustments and modifications to enhance their performance. These endeavors underscore the dynamic nature of research and the iterative process required to propel cancer vaccines toward their full therapeutic potential.

Future advancement of the clinical efficacy of therapeutic cancer vaccines is concentrated in several key areas, including identifying immunogenic neoantigens, optimizing vectors and delivery platforms, and surmounting the immunosuppressive TME. Next-generation sequencing, increased computing power, and advanced algorithms have significantly enabled identifying highly immunogenic neoantigens. Ongoing research is dedicated to refining vaccine technologies, including exploring diverse expression formats, improving co-stimulation components, and identifying suitable prime-boost approaches. Optimally selecting a delivery system is crucial to amplify antigen immunogenicity and facilitate the entry of the antigen and an activation signal into APCs. Strategically incorporating immune checkpoint inhibitors and other treatment regimens is another avenue for exploration. These combined approaches can counteract the immunosuppressive milieu of the TME and mitigate immunotherapy resistance development. However, the precise composition of combination therapies, and their sequence and dosage, require further investigation and refinement.

Establishing a tumor-specific cytotoxic T cell response remains a pivotal challenge in cancer immunotherapy. The overarching objective of these multifaceted strategies involves selecting a treatment regimen that stimulates effective, durable, and tumor-specific immunity in patients with cancer and prolongs their survival through cancer vaccines. The convergence of innovative approaches, strategic candidate selection, and refined administration protocols hold the potential to revolutionize cancer treatment, paving the way for a new era of therapeutic cancer vaccines. We will witness the successful emergence of numerous therapeutic cancer vaccines with ongoing advancements.

## Data Availability

All relevant data are within the manuscript and its additional files.

## References

[1] Sung, H. et al. Global Cancer Statistics 2020: GLOBOCAN Estimates of Incidence and Mortality Worldwide for 36 Cancers in 185 Countries. *CA Cancer J. Clin.* **71**, 209–249 (2021). 33538338 10.3322/caac.21660

[2] Mirzayans, R. et al. What Are the Reasons for Continuing Failures in Cancer Therapy? Are Misleading/Inappropriate Preclinical Assays to Be Blamed? Might Some Modern Therapies Cause More Harm than Benefit? *Int. J. Mol. Sci*. **23** (2022). 10.3390/ijms232113217 PMC9655591 36362004

[3] Schreiber, R. D. et al. Cancer immunoediting: integrating immunity’s roles in cancer suppression and promotion. *Science* **331**, 1565–1570 (2011). 21436444 10.1126/science.1203486

[4] Zhang, Y. et al. The history and advances in cancer immunotherapy: understanding the characteristics of tumor-infiltrating immune cells and their therapeutic implications. *Cell. Mol. Immunol.* **17**, 807–821 (2020). 32612154 10.1038/s41423-020-0488-6 PMC7395159

[5] Feola, S. et al. Oncolytic ImmunoViroTherapy: A long history of crosstalk between viruses and immune system for cancer treatment. *Pharmacol. Ther.* **236**, 108103 (2022). 34954301 10.1016/j.pharmthera.2021.108103

[6] Liu, L. et al. Engineering chimeric antigen receptor T cells for solid tumour therapy. *Clin. Transl. Med.* **12**, e1141 (2022). 36495108 10.1002/ctm2.1141 PMC9736813

[7] Haslam, A. et al. Estimation of the Percentage of US Patients With Cancer Who Are Eligible for and Respond to Checkpoint Inhibitor Immunotherapy Drugs. *JAMA Netw*. *Open* **2**, e192535–e192535 (2019). 10.1001/jamanetworkopen.2019.2535 PMC6503493 31050774

[8] Oladejo, M. et al. Synergistic potential of immune checkpoint inhibitors and therapeutic cancer vaccines. *Semin. Cancer Biol.* **88**, 81–95 (2023). 36526110 10.1016/j.semcancer.2022.12.003

[9] Saxena, M. et al. Therapeutic cancer vaccines. *Nat. Rev. Cancer* **21**, 360–378 (2021). 33907315 10.1038/s41568-021-00346-0

[10] Sellars, M. C. et al. Cancer vaccines: Building a bridge over troubled waters. *Cell* **185**, 2770–2788 (2022). 35835100 10.1016/j.cell.2022.06.035 PMC9555301

[11] Lin, M. J. et al. Cancer vaccines: the next immunotherapy frontier. *Nat. Cancer* **3**, 911–926 (2022). 35999309 10.1038/s43018-022-00418-6

[12] Leko, V. et al. Identifying and Targeting Human Tumor Antigens for T Cell-Based Immunotherapy of Solid Tumors. *Cancer Cell* **38**, 454–472 (2020). 32822573 10.1016/j.ccell.2020.07.013 PMC7737225

[13] Jou, J. et al. The Changing Landscape of Therapeutic Cancer Vaccines-Novel Platforms and Neoantigen Identification. *Clin. Cancer Res.* **27**, 689–703 (2021). 33122346 10.1158/1078-0432.CCR-20-0245

[14] Romano, E. et al. MART-1 peptide vaccination plus IMP321 (LAG-3Ig fusion protein) in patients receiving autologous PBMCs after lymphodepletion: results of a Phase I trial. *J. Transl. Med.* **12**, 97 (2014). 24726012 10.1186/1479-5876-12-97 PMC4021605

[15] Disis, M. L. N. et al. Safety and Outcomes of a Plasmid DNA Vaccine Encoding the ERBB2 Intracellular Domain in Patients With Advanced-Stage ERBB2-Positive Breast Cancer: A Phase 1 Nonrandomized Clinical Trial. *JAMA Oncol.* **9**, 71–78 (2023). 36326756 10.1001/jamaoncol.2022.5143 PMC9634596

[16] Fan, C. et al. Cancer/testis antigens: from serology to mRNA cancer vaccine. *Semin. Cancer Biol.* **76**, 218–231 (2021). 33910064 10.1016/j.semcancer.2021.04.016

[17] Schooten, E. et al. MAGE-A antigens as targets for cancer immunotherapy. *Cancer Treat. Rev.* **67**, 54–62 (2018). 29763778 10.1016/j.ctrv.2018.04.009

[18] Berman, T. A. et al. Human papillomavirus in cervical cancer and oropharyngeal cancer: One cause, two diseases. *Cancer* **123**, 2219–2229 (2017). 28346680 10.1002/cncr.30588

[19] Peng, M. et al. Neoantigen vaccine: an emerging tumor immunotherapy. *Mol. Cancer* **18**, 128 (2019). 31443694 10.1186/s12943-019-1055-6 PMC6708248

[20] Kantoff, P. W. et al. Sipuleucel-T immunotherapy for castration-resistant prostate cancer. *N. Engl. J. Med.* **363**, 411–422 (2010). 20818862 10.1056/NEJMoa1001294

[21] Wargowski, E. et al. Prime-boost vaccination targeting prostatic acid phosphatase (PAP) in patients with metastatic castration-resistant prostate cancer (mCRPC) using Sipuleucel-T and a DNA vaccine. *J. Immunother. Cancer* **6**, 21 (2018). 29534736 10.1186/s40425-018-0333-y PMC5850960

[22] Sahin, U. et al. An RNA vaccine drives immunity in checkpoint-inhibitor-treated melanoma. *Nature* **585**, 107–112 (2020). 32728218 10.1038/s41586-020-2537-9

[23] Moderna’s therapeutics: KRAS vaccine (mRNA-5671). https://investors.modernatx.com/events-and-presentations/presentations/presentation-details/2021/mRNA-5671/default.aspx (2021).

[24] Kreutmair, S. et al. First-in-human study of WT1 recombinant protein vaccination in elderly patients with AML in remission: a single-center experience. *Cancer Immunol. Immunother.* **71**, 2913–2928 (2022). 35476127 10.1007/s00262-022-03202-8 PMC9588470

[25] Alsalloum, A. et al. The Melanoma-Associated Antigen Family A (MAGE-A): A Promising Target for Cancer Immunotherapy? *Cancers (Basel)* **15**, 1779 (2023). 36980665 10.3390/cancers15061779 PMC10046478

[26] Dreno, B. et al. MAGE-A3 immunotherapeutic as adjuvant therapy for patients with resected, MAGE-A3-positive, stage III melanoma (DERMA): a double-blind, randomised, placebo-controlled, phase 3 trial. *Lancet Oncol.* **19**, 916–929 (2018). 29908991 10.1016/S1470-2045(18)30254-7

[27] Vansteenkiste, J. F. et al. Efficacy of the MAGE-A3 cancer immunotherapeutic as adjuvant therapy in patients with resected MAGE-A3-positive non-small-cell lung cancer (MAGRIT): a randomised, double-blind, placebo-controlled, phase 3 trial. *Lancet Oncol.* **17**, 822–835 (2016). 27132212 10.1016/S1470-2045(16)00099-1

[28] Duperret, E. K. et al. A Designer Cross-reactive DNA Immunotherapeutic Vaccine that Targets Multiple MAGE-A Family Members Simultaneously for Cancer Therapy. *Clin*. *Cancer Res* **24**, 6015–6027 (2018). 10.1158/1078-0432.CCR-18-1013 PMC6319943 30262507

[29] Butts, C. et al. Tecemotide (L-BLP25) versus placebo after chemoradiotherapy for stage III non-small-cell lung cancer (START): a randomised, double-blind, phase 3 trial. *Lancet Oncol.* **15**, 59–68 (2014). 24331154 10.1016/S1470-2045(13)70510-2

[30] Mitchell, P. et al. Tecemotide in unresectable stage III non-small-cell lung cancer in the phase III START study: updated overall survival and biomarker analyses. *Ann. Oncol.* **26**, 1134–1142 (2015). 25722382 10.1093/annonc/mdv104

[31] Harbeck, N. et al. Breast cancer. *Lancet* **389**, 1134–1150 (2017). 27865536 10.1016/S0140-6736(16)31891-8

[32] Mittendorf, E. A. et al. Efficacy and Safety Analysis of Nelipepimut-S Vaccine to Prevent Breast Cancer Recurrence: A Randomized, Multicenter, Phase III Clinical Trial. *Clin. Cancer Res.* **25**, 4248–4254 (2019). 31036542 10.1158/1078-0432.CCR-18-2867

[33] Clifton, G. T. et al. Results of a Randomized Phase IIb Trial of Nelipepimut-S + Trastuzumab versus Trastuzumab to Prevent Recurrences in Patients with High-Risk HER2 Low-Expressing Breast Cancer. *Clin. Cancer Res.* **26**, 2515–2523 (2020). 32071118 10.1158/1078-0432.CCR-19-2741 PMC8771534

[34] Cui, X. et al. Epstein Barr Virus: Development of Vaccines and Immune Cell Therapy for EBV-Associated Diseases. *Front. Immunol.* **12**, 734471 (2021). 34691042 10.3389/fimmu.2021.734471 PMC8532523

[35] Taylor, G. S. et al. A recombinant modified vaccinia ankara vaccine encoding Epstein-Barr Virus (EBV) target antigens: a phase I trial in UK patients with EBV-positive cancer. *Clin. Cancer Res.* **20**, 5009–5022 (2014). 25124688 10.1158/1078-0432.CCR-14-1122-T PMC4340506

[36] Chia, W. K. et al. A phase II study evaluating the safety and efficacy of an adenovirus-ΔLMP1-LMP2 transduced dendritic cell vaccine in patients with advanced metastatic nasopharyngeal carcinoma. *Ann. Oncol.* **23**, 997–1005 (2012). 21821548 10.1093/annonc/mdr341 PMC3314324

[37] Bu, W. et al. Immunization with Components of the Viral Fusion Apparatus Elicits Antibodies That Neutralize Epstein-Barr Virus in B Cells and Epithelial Cells. *Immunity* **50**, 1305–1316.e1306 (2019). 30979688 10.1016/j.immuni.2019.03.010 PMC6660903

[38] Grunwitz, C. et al. HPV16 RNA-LPX vaccine mediates complete regression of aggressively growing HPV-positive mouse tumors and establishes protective T cell memory. *Oncoimmunology* **8**, e1629259 (2019). 31428528 10.1080/2162402X.2019.1629259 PMC6685602

[39] Youn, J. W. et al. Pembrolizumab plus GX-188E therapeutic DNA vaccine in patients with HPV-16-positive or HPV-18-positive advanced cervical cancer: interim results of a single-arm, phase 2 trial. *Lancet Oncol.* **21**, 1653–1660 (2020). 33271094 10.1016/S1470-2045(20)30486-1

[40] Ott, P. et al. An immunogenic personal neoantigen vaccine for patients with melanoma. *Nature* **547**, 217–221 (2017). 28678778 10.1038/nature22991 PMC5577644

[41] Lancaster, J. N. et al. Central tolerance is impaired in the middle-aged thymic environment. *Aging Cell* **21**, e13624 (2022). 35561351 10.1111/acel.13624 PMC9197411

[42] Schumacher, T. et al. A vaccine targeting mutant IDH1 induces antitumour immunity. *Nature* **512**, 324–327 (2014). 25043048 10.1038/nature13387

[43] Wang, Y. et al. Gene fusion neoantigens: Emerging targets for cancer immunotherapy. *Cancer Lett.* **506**, 45–54 (2021). 33675984 10.1016/j.canlet.2021.02.023

[44] Zhou, C. et al. Systematically Characterizing A-to-I RNA Editing Neoantigens in Cancer. *Front. Oncol.* **10**, 593989 (2020). 33363023 10.3389/fonc.2020.593989 PMC7758481

[45] De Mattos-Arruda, L. et al. Neoantigen prediction and computational perspectives towards clinical benefit: recommendations from the ESMO Precision Medicine Working Group. *Ann. Oncol.* **31**, 978–990 (2020). 32610166 10.1016/j.annonc.2020.05.008 PMC7885309

[46] Xie, N. et al. Neoantigens: promising targets for cancer therapy. *Signal Transduct. Target Ther.* **8**, 9 (2023). 36604431 10.1038/s41392-022-01270-x PMC9816309

[47] Chen, F. et al. Neoantigen identification strategies enable personalized immunotherapy in refractory solid tumors. *J. Clin. Invest* **129**, 2056–2070 (2019). 30835255 10.1172/JCI99538 PMC6486339

[48] Lang, F. et al. Identification of neoantigens for individualized therapeutic cancer vaccines. *Nat. Rev. Drug Discov.* **21**, 261–282 (2022). 35105974 10.1038/s41573-021-00387-y PMC7612664

[49] Zhou, C. et al. Toward in silico Identification of Tumor Neoantigens in Immunotherapy. *Trends Mol. Med.* **25**, 980–992 (2019). 31494024 10.1016/j.molmed.2019.08.001

[50] Yadav, M. et al. Predicting immunogenic tumour mutations by combining mass spectrometry and exome sequencing. *Nature* **515**, 572–576 (2014). 25428506 10.1038/nature14001

[51] Abelin, J. G. et al. Defining HLA-II Ligand Processing and Binding Rules with Mass Spectrometry Enhances Cancer Epitope Prediction. *Immunity* **51**, 766–779.e717 (2019). 31495665 10.1016/j.immuni.2019.08.012

[52] Bulik-Sullivan, B. et al. Deep learning using tumor HLA peptide mass spectrometry datasets improves neoantigen identification. *Nat. Biotechnol.* **37**, 55–63 (2018). 10.1038/nbt.4313 30556813

[53] Zhou, C. et al. pTuneos: prioritizing tumor neoantigens from next-generation sequencing data. *Genome Med.* **11**, 67 (2019). 31666118 10.1186/s13073-019-0679-x PMC6822339

[54] Hundal, J. et al. pVAC-Seq: A genome-guided in silico approach to identifying tumor neoantigens. *Genome Med.* **8**, 11 (2016). 26825632 10.1186/s13073-016-0264-5 PMC4733280

[55] Tappeiner, E. et al. TIminer: NGS data mining pipeline for cancer immunology and immunotherapy. *Bioinformatics* **33**, 3140–3141 (2017). 28633385 10.1093/bioinformatics/btx377 PMC5870678

[56] Liu, C. et al. ProGeo-Neo v2.0: A One-Stop Software for Neoantigen Prediction and Filtering Based on the Proteogenomics Strategy. *Genes (Basel)* **13**, 783 (2022). 35627168 10.3390/genes13050783 PMC9141370

[57] Zhang, J. et al. INTEGRATE-neo: a pipeline for personalized gene fusion neoantigen discovery. *Bioinformatics* **33**, 555–557 (2017). 27797777 10.1093/bioinformatics/btw674 PMC5408800

[58] Robinson, J. et al. The IPD and IMGT/HLA database: allele variant databases. *Nucleic Acids Res.* **43**, D423–D431 (2015). 25414341 10.1093/nar/gku1161 PMC4383959

[59] Szolek, A. et al. OptiType: precision HLA typing from next-generation sequencing data. *Bioinformatics* **30**, 3310–3316 (2014). 25143287 10.1093/bioinformatics/btu548 PMC4441069

[60] Matey-Hernandez, M. L. et al. Benchmarking the HLA typing performance of Polysolver and Optitype in 50 Danish parental trios. *BMC Bioinforma.* **19**, 239 (2018). 10.1186/s12859-018-2239-6 PMC6019707 29940840

[61] Ka, S. et al. HLAscan: genotyping of the HLA region using next-generation sequencing data. *BMC Bioinforma.* **18**, 258 (2017). 10.1186/s12859-017-1671-3 PMC5427585 28499414

[62] Bai, Y. et al. PHLAT: Inference of High-Resolution HLA Types from RNA and Whole Exome Sequencing. *Methods Mol. Biol.* **1802**, 193–201 (2018). 29858810 10.1007/978-1-4939-8546-3_13

[63] Boegel, S. et al. HLA typing from RNA-Seq sequence reads. *Genome Med.* **4**, 102 (2012). 23259685 10.1186/gm403 PMC4064318

[64] Dilthey, A. T. et al. High-Accuracy HLA Type Inference from Whole-Genome Sequencing Data Using Population Reference Graphs. *PLoS Comput. Biol.* **12**, e1005151 (2016). 27792722 10.1371/journal.pcbi.1005151 PMC5085092

[65] Lee, H. et al. Kourami: graph-guided assembly for novel human leukocyte antigen allele discovery. *Genome Biol.* **19**, 16 (2018). 29415772 10.1186/s13059-018-1388-2 PMC5804087

[66] Zaretsky, J. M. et al. Mutations associated with acquired resistance to PD-1 blockade in melanoma. *N. Engl. J. Med.* **375**, 819–829 (2016). 27433843 10.1056/NEJMoa1604958 PMC5007206

[67] Sahin, U. et al. Personalized RNA mutanome vaccines mobilize poly-specific therapeutic immunity against cancer. *Nature* **547**, 222–226 (2017). 28678784 10.1038/nature23003

[68] Leone, P. et al. MHC class I antigen processing and presenting machinery: organization, function, and defects in tumor cells. *J. Natl Cancer Inst.* **105**, 1172–1187 (2013). 23852952 10.1093/jnci/djt184

[69] Robbins, P. F. et al. Mining exomic sequencing data to identify mutated antigens recognized by adoptively transferred tumor-reactive T cells. *Nat. Med.* **19**, 747–752 (2013). 23644516 10.1038/nm.3161 PMC3757932

[70] Lee, M. Y. et al. Antigen processing and presentation in cancer immunotherapy. *J Immunother Cancer* **8**, e001111 (2020). 32859742 10.1136/jitc-2020-001111 PMC7454179

[71] Calis, J. J. et al. Role of peptide processing predictions in T cell epitope identification: contribution of different prediction programs. *Immunogenetics* **67**, 85–93 (2015). 25475908 10.1007/s00251-014-0815-0 PMC4297296

[72] Lam, T. H. et al. TAP Hunter: a SVM-based system for predicting TAP ligands using local description of amino acid sequence. *Immunome. Res.* **6**, S6 (2010). 20875157 10.1186/1745-7580-6-S1-S6 PMC2946784

[73] Pishesha, N. et al. A guide to antigen processing and presentation. *Nat. Rev. Immunol.* **22**, 751–764 (2022). 35418563 10.1038/s41577-022-00707-2

[74] Reynisson, B. et al. NetMHCpan-4.1 and NetMHCIIpan-4.0: improved predictions of MHC antigen presentation by concurrent motif deconvolution and integration of MS MHC eluted ligand data. *Nucleic Acids Res.* **48**, W449–w454 (2020). 32406916 10.1093/nar/gkaa379 PMC7319546

[75] Zhao, W. et al. Systematically benchmarking peptide-MHC binding predictors: From synthetic to naturally processed epitopes. *PLoS Comput. Biol.* **14**, e1006457 (2018). 30408041 10.1371/journal.pcbi.1006457 PMC6224037

[76] O’Donnell, T. J. et al. MHCflurry: Open-Source Class I MHC Binding Affinity Prediction. *Cell Syst.* **7**, 129–132.e124 (2018). 29960884 10.1016/j.cels.2018.05.014

[77] Mei, S. et al. A comprehensive review and performance evaluation of bioinformatics tools for HLA class I peptide-binding prediction. *Brief. Bioinform* **21**, 1119–1135 (2020). 31204427 10.1093/bib/bbz051 PMC7373177

[78] You, R. et al. DeepMHCII: a novel binding core-aware deep interaction model for accurate MHC-II peptide binding affinity prediction. *Bioinformatics* **38**, i220–i228 (2022). 35758790 10.1093/bioinformatics/btac225 PMC9235502

[79] Garde, C. et al. Improved peptide-MHC class II interaction prediction through integration of eluted ligand and peptide affinity data. *Immunogenetics* **71**, 445–454 (2019). 31183519 10.1007/s00251-019-01122-z

[80] Harndahl, M. et al. Peptide-MHC class I stability is a better predictor than peptide affinity of CTL immunogenicity. *Eur. J. Immunol.* **42**, 1405–1416 (2012). 22678897 10.1002/eji.201141774

[81] Rasmussen, M. et al. Pan-Specific Prediction of Peptide-MHC Class I Complex Stability, a Correlate of T Cell Immunogenicity. *J. Immunol.* **197**, 1517–1524 (2016). 27402703 10.4049/jimmunol.1600582 PMC4976001

[82] Blaha, D. T. et al. High-Throughput Stability Screening of Neoantigen/HLA Complexes Improves Immunogenicity Predictions. *Cancer Immunol. Res.* **7**, 50–61 (2019). 30425106 10.1158/2326-6066.CIR-18-0395 PMC6324732

[83] Glanville, J. et al. Identifying specificity groups in the T cell receptor repertoire. *Nature* **547**, 94–98 (2017). 28636589 10.1038/nature22976 PMC5794212

[84] Sidhom, J. W. et al. DeepTCR is a deep learning framework for revealing sequence concepts within T-cell repertoires. *Nat. Commun.* **12**, 1605 (2021). 33707415 10.1038/s41467-021-21879-w PMC7952906

[85] Chronister, W. D. et al. TCRMatch: Predicting T-Cell Receptor Specificity Based on Sequence Similarity to Previously Characterized Receptors. *Front. Immunol.* **12**, 640725 (2021). 33777034 10.3389/fimmu.2021.640725 PMC7991084

[86] Montemurro, A. et al. NetTCR-2.1: Lessons and guidance on how to develop models for TCR specificity predictions. *Front. Immunol.* **13**, 1055151 (2022). 36561755 10.3389/fimmu.2022.1055151 PMC9763291

[87] Xu, Z. et al. DLpTCR: an ensemble deep learning framework for predicting immunogenic peptide recognized by T cell receptor. *Brief. Bioinform.* **22**, bbab335 (2021). 34415016 10.1093/bib/bbab335

[88] Pham, M. N. et al. epiTCR: a highly sensitive predictor for TCR-peptide binding. *Bioinformatics* **39**, btad284 (2023). 37094220 10.1093/bioinformatics/btad284 PMC10159657

[89] Weber, A. et al. TITAN: T-cell receptor specificity prediction with bimodal attention networks. *Bioinformatics* **37**, i237–i244 (2021). 34252922 10.1093/bioinformatics/btab294 PMC8275323

[90] Gao, Y. et al. Pan-Peptide Meta Learning for T-cell receptor–antigen binding recognition. *Nat. Mach. Intell.* **5**, 236–249 (2023). 10.1038/s42256-023-00619-3

[91] O’Donnell, T. J. et al. MHCflurry 2.0: Improved Pan-Allele Prediction of MHC Class I-Presented Peptides by Incorporating Antigen Processing. *Cell Syst.* **11**, 42–48.e47 (2020). 32711842 10.1016/j.cels.2020.06.010

[92] Zhou, Z. et al. TSNAD v2.0: A one-stop software solution for tumor-specific neoantigen detection. *Comput Struct. Biotechnol. J.* **19**, 4510–4516 (2021). 34471496 10.1016/j.csbj.2021.08.016 PMC8385119

[93] Hundal, J. et al. pVACtools: A Computational Toolkit to Identify and Visualize Cancer Neoantigens. *Cancer Immunol. Res.* **8**, 409–420 (2020). 31907209 10.1158/2326-6066.CIR-19-0401 PMC7056579

[94] Gros, A. et al. PD-1 identifies the patient-specific CD8^+^ tumor-reactive repertoire infiltrating human tumors. *J. Clin. Invest.* **124**, 2246–2259 (2014). 24667641 10.1172/JCI73639 PMC4001555

[95] Gubin, M. M. et al. Tumor neoantigens: building a framework for personalized cancer immunotherapy. *J. Clin. Invest* **125**, 3413–3421 (2015). 26258412 10.1172/JCI80008 PMC4588307

[96] Kreiter, S. et al. Mutant MHC class II epitopes drive therapeutic immune responses to cancer. *Nature* **520**, 692–696 (2015). 25901682 10.1038/nature14426 PMC4838069

[97] Ott, P. A. et al. A Phase Ib Trial of Personalized Neoantigen Therapy Plus Anti-PD-1 in Patients with Advanced Melanoma, Non-small. *Cell Lung Cancer, or Bladder Cancer. Cell* **183**, 347–362.e324 (2020). 33064988 10.1016/j.cell.2020.08.053

[98] Moderna and Merck Announce mRNA-4157/V940, an Investigational Personalized mRNA Cancer Vaccine, in Combination With KEYTRUDA(R) (pembrolizumab), was Granted Breakthrough Therapy Designation by the FDA for Adjuvant Treatment of Patients With High-Risk Melanoma Following CompleteResection. https://investors.modernatx.com/news/news-details/2023/Moderna-and-Merck-Announce-mRNA-4157V940-an-Investigational-Personalized-mRNA-Cancer-Vaccine-in-Combination-With-KEYTRUDAR-pembrolizumab-was-Granted-Breakthrough-Therapy-Designation-by-the-FDA-for-Adjuvant-Treatment-of-Patients-With-High-Risk-Melanom/default.aspx (2023).

[99] Liu, S. et al. A DNA nanodevice-based vaccine for cancer immunotherapy. *Nat. Mater.* **20**, 421–430 (2021). 32895504 10.1038/s41563-020-0793-6

[100] Ori, D. et al. Cytosolic nucleic acid sensors and innate immune regulation. *Int. Rev. Immunol.* **36**, 74–88 (2017). 28333574 10.1080/08830185.2017.1298749

[101] Nguyen-Hoai, T. et al. Gene Gun Her2/neu DNA Vaccination: Evaluation of Vaccine Efficacy in a Syngeneic Her2/neu Mouse Tumor Model. *Methods Mol. Biol.* **2521**, 129–154 (2022). 35732996 10.1007/978-1-0716-2441-8_7

[102] Elizaga, M. L. et al. Safety and tolerability of HIV-1 multiantigen pDNA vaccine given with IL-12 plasmid DNA via electroporation, boosted with a recombinant vesicular stomatitis virus HIV Gag vaccine in healthy volunteers in a randomized, controlled clinical trial. *PLoS One* **13**, e0202753 (2018). 30235286 10.1371/journal.pone.0202753 PMC6147413

[103] Nishimura, K. et al. Suppression of Peritoneal Fibrosis by Sonoporation of Hepatocyte Growth Factor Gene-Encoding Plasmid DNA in Mice. *Pharmaceutics* **13**, 115 (2021). 33477422 10.3390/pharmaceutics13010115 PMC7829751

[104] Suschak, J. J. et al. Advancements in DNA vaccine vectors, non-mechanical delivery methods, and molecular adjuvants to increase immunogenicity. *Hum. Vaccin. Immunother.* **13**, 2837–2848 (2017). 28604157 10.1080/21645515.2017.1330236 PMC5718814

[105] (2021) 10 Breakthrough Technologies 2021.

[106] He, Q. et al. mRNA cancer vaccines: Advances, trends and challenges. *Acta Pharm. Sin. B* **12**, 2969–2989 (2022). 35345451 10.1016/j.apsb.2022.03.011 PMC8942458

[107] Pardi, N. et al. mRNA vaccines — a new era in vaccinology. *Nat. Rev. Drug Discov.* **17**, 261–279 (2018). 29326426 10.1038/nrd.2017.243 PMC5906799

[108] Gong, H. et al. Integrated mRNA sequence optimization using deep learning. *Brief. Bioinform.* **24**, bbad001 (2023). 36642413 10.1093/bib/bbad001 PMC9851294

[109] Zhang, H. et al. Algorithm for Optimized mRNA Design Improves Stability and Immunogenicity. *Nature* **621**, 396–403 (2023). 37130545 10.1038/s41586-023-06127-z PMC10499610

[110] Crommelin, D. J. A. et al. Addressing the Cold Reality of mRNA Vaccine Stability. *J. Pharm. Sci.* **110**, 997–1001 (2021). 33321139 10.1016/j.xphs.2020.12.006 PMC7834447

[111] Hou, X. et al. Lipid nanoparticles for mRNA delivery. *Nat. Rev. Mater.* **6**, 1078–1094 (2021). 34394960 10.1038/s41578-021-00358-0 PMC8353930

[112] Jarzebska, N. T. et al. Protamine-Based Strategies for RNA Transfection. *Pharmaceutics* **13**, 877 (2021). 34198550 10.3390/pharmaceutics13060877 PMC8231816

[113] Kübler, H. et al. Self-adjuvanted mRNA vaccination in advanced prostate cancer patients: a first-in-man phase I/IIa study. *J. Immunother. Cancer* **3**, 26 (2015). 26082837 10.1186/s40425-015-0068-y PMC4468959

[114] Cafri, G. et al. mRNA vaccine-induced neoantigen-specific T cell immunity in patients with gastrointestinal cancer. *J. Clin. Invest.* **130**, 5976–5988 (2020). 33016924 10.1172/JCI134915 PMC7598064

[115] Personalized Cancer Vaccine Plus Atezolizumab Shows Clinical Activity in Patients With Advanced Solid Tumors. https://www.aacr.org/about-the-aacr/newsroom/news-releases/personalized-cancer-vaccine-plus-atezolizumab-shows-clinical-activity-in-patients-with-advanced-solid-tumors/ (2020).

[116] Rojas, L. A. et al. Personalized RNA neoantigen vaccines stimulate T cells in pancreatic cancer. *Nature* **618**, 144–150 (2023). 37165196 10.1038/s41586-023-06063-y PMC10171177

[117] Major agreement to deliver new cancer vaccine trials. https://www.gov.uk/government/news/major-agreement-to-deliver-new-cancer-vaccine-trials (2023).

[118] Zwaveling, S. et al. Established human papillomavirus type 16-expressing tumors are effectively eradicated following vaccination with long peptides. *J. Immunol.* **169**, 350–358 (2002). 12077264 10.4049/jimmunol.169.1.350

[119] Melief, C. J. et al. Immunotherapy of established (pre)malignant disease by synthetic long peptide vaccines. *Nat. Rev. Cancer* **8**, 351–360 (2008). 18418403 10.1038/nrc2373

[120] Chen, X. et al. Personalized neoantigen vaccination with synthetic long peptides: recent advances and future perspectives. *Theranostics* **10**, 6011–6023 (2020). 32483434 10.7150/thno.38742 PMC7255011

[121] Sobhani, N. et al. Therapeutic cancer vaccines: From biological mechanisms and engineering to ongoing clinical trials. *Cancer Treat. Rev.* **109**, 102429 (2022). 35759856 10.1016/j.ctrv.2022.102429 PMC9217071

[122] Liu, W. et al. Peptide-based therapeutic cancer vaccine: Current trends in clinical application. *Cell Prolif.* **54**, e13025 (2021). 33754407 10.1111/cpr.13025 PMC8088465

[123] Parmiani, G. et al. Opposite immune functions of GM-CSF administered as vaccine adjuvant in cancer patients. *Ann. Oncol.* **18**, 226–232 (2007). 17116643 10.1093/annonc/mdl158

[124] Lawson, D. H. et al. Randomized, Placebo-Controlled, Phase III Trial of Yeast-Derived Granulocyte-Macrophage Colony-Stimulating Factor (GM-CSF) Versus Peptide Vaccination Versus GM-CSF Plus Peptide Vaccination Versus Placebo in Patients With No Evidence of Disease After Complete Surgical Resection of Locally Advanced and/or Stage IV Melanoma: A Trial of the Eastern Cooperative Oncology Group-American College of Radiology Imaging Network Cancer Research Group (E4697). *J. Clin. Oncol.* **33**, 4066–4076 (2015). 26351350 10.1200/JCO.2015.62.0500 PMC4669592

[125] Hilf, N. et al. Actively personalized vaccination trial for newly diagnosed glioblastoma. *Nature* **565**, 240–245 (2019). 30568303 10.1038/s41586-018-0810-y

[126] Lynn, G. M. et al. Peptide-TLR-7/8a conjugate vaccines chemically programmed for nanoparticle self-assembly enhance CD8 T-cell immunity to tumor antigens. *Nat. Biotechnol.* **38**, 320–332 (2020). 31932728 10.1038/s41587-019-0390-x PMC7065950

[127] Patel, S. P. et al. Phase I/II trial of a long peptide vaccine (LPV7) plus toll-like receptor (TLR) agonists with or without incomplete Freund’s adjuvant (IFA) for resected high-risk melanoma. *J Immunother Cancer* **9**, e003220 (2021). 34413169 10.1136/jitc-2021-003220 PMC8378357

[128] Yamada, A. et al. Next-generation peptide vaccines for advanced cancer. *Cancer Sci.* **104**, 15–21 (2013). 23107418 10.1111/cas.12050 PMC7657262

[129] Najafi, S. et al. Advances in dendritic cell vaccination therapy of cancer. *Biomed. Pharmacother.* **164**, 114954 (2023). 37257227 10.1016/j.biopha.2023.114954

[130] Keskin, D. B. et al. Neoantigen vaccine generates intratumoral T cell responses in phase Ib glioblastoma trial. *Nature* **565**, 234–239 (2019). 30568305 10.1038/s41586-018-0792-9 PMC6546179

[131] Steele, J. C. et al. Phase I/II trial of a dendritic cell vaccine transfected with DNA encoding melan A and gp100 for patients with metastatic melanoma. *Gene Ther.* **18**, 584–593 (2011). 21307889 10.1038/gt.2011.1

[132] Mitchell, D. A. et al. Tetanus toxoid and CCL3 improve dendritic cell vaccines in mice and glioblastoma patients. *Nature* **519**, 366–369 (2015). 25762141 10.1038/nature14320 PMC4510871

[133] Van Driessche, A. et al. Clinical-grade manufacturing of autologous mature mRNA-electroporated dendritic cells and safety testing in acute myeloid leukemia patients in a phase I dose-escalation clinical trial. *Cytotherapy* **11**, 653–668 (2009). 19530029 10.1080/14653240902960411

[134] Tanyi, J. L. et al. Personalized cancer vaccine effectively mobilizes antitumor T cell immunity in ovarian cancer. *Sci. Transl. Med.* **10**, eaao5931 (2018). 29643231 10.1126/scitranslmed.aao5931

[135] Ebrahimi-Nik, H. et al. CD11c(+) MHCII(lo) GM-CSF-bone marrow-derived dendritic cells act as antigen donor cells and as antigen presenting cells in neoepitope-elicited tumor immunity against a mouse fibrosarcoma. *Cancer Immunol. Immunother.* **67**, 1449–1459 (2018). 30030558 10.1007/s00262-018-2202-4 PMC6132860

[136] Ding, Z. et al. Personalized neoantigen pulsed dendritic cell vaccine for advanced lung cancer. *Signal Transduct. Target Ther.* **6**, 26 (2021). 33473101 10.1038/s41392-020-00448-5 PMC7817684

[137] Liau, L. M. et al. Association of Autologous Tumor Lysate-Loaded Dendritic Cell Vaccination With Extension of Survival Among Patients With Newly Diagnosed and Recurrent Glioblastoma: A Phase 3 Prospective Externally Controlled Cohort Trial. *JAMA Oncol* **9**, 112–121 (2022). 10.1001/jamaoncol.2022.5370 PMC9673026 36394838

[138] Yu, Z. et al. HLA-A2.1-restricted ECM1-derived epitope LA through DC cross-activation priming CD8(+) T and NK cells: a novel therapeutic tumour vaccine. *J. Hematol. Oncol.* **14**, 71 (2021). 33910591 10.1186/s13045-021-01081-7 PMC8082934

[139] Schlitzer, A. et al. Identification of CCR9- murine plasmacytoid DC precursors with plasticity to differentiate into conventional DCs. *Blood* **117**, 6562–6570 (2011). 21508410 10.1182/blood-2010-12-326678

[140] Reizis, B. Plasmacytoid Dendritic Cells: Development, Regulation, and Function. *Immunity* **50**, 37–50 (2019). 30650380 10.1016/j.immuni.2018.12.027 PMC6342491

[141] Kvedaraite, E. et al. Human dendritic cells in cancer. *Sci. Immunol.* **7**, eabm9409 (2022). 35363544 10.1126/sciimmunol.abm9409

[142] Than, U. T. T. et al. Induction of Antitumor Immunity by Exosomes Isolated from Cryopreserved Cord Blood Monocyte-Derived Dendritic Cells. *Int. J. Mol. Sci.* **21**, 1834 (2020). 32155869 10.3390/ijms21051834 PMC7084404

[143] Thordardottir, S. et al. Hematopoietic stem cell-derived myeloid and plasmacytoid DC-based vaccines are highly potent inducers of tumor-reactive T cell and NK cell responses ex vivo. *Oncoimmunology* **6**, e1285991 (2017). 28405517 10.1080/2162402X.2017.1285991 PMC5384421

[144] Förster, R. et al. CCR7 and its ligands: balancing immunity and tolerance. *Nat. Rev. Immunol.* **8**, 362–371 (2008). 18379575 10.1038/nri2297

[145] Xu, J. et al. CCR7 Mediated Mimetic Dendritic Cell Vaccine Homing in Lymph Node for Head and Neck Squamous Cell Carcinoma Therapy. *Adv. Sci. (Weinh.)* **10**, e2207017 (2023). 37092579 10.1002/advs.202207017 PMC10265089

[146] Hadeiba, H. et al. Plasmacytoid dendritic cells transport peripheral antigens to the thymus to promote central tolerance. *Immunity* **36**, 438–450 (2012). 22444632 10.1016/j.immuni.2012.01.017 PMC3315699

[147] Kreiter, S. et al. FLT3 ligand enhances the cancer therapeutic potency of naked RNA vaccines. *Cancer Res.* **71**, 6132–6142 (2011). 21816907 10.1158/0008-5472.CAN-11-0291

[148] Huang, L. et al. Engineered exosomes as an in situ DC-primed vaccine to boost antitumor immunity in breast cancer. *Mol. Cancer* **21**, 45 (2022). 35148751 10.1186/s12943-022-01515-x PMC8831689

[149] Creusot, R. J. et al. Lymphoid-tissue-specific homing of bone-marrow-derived dendritic cells. *Blood* **113**, 6638–6647 (2009). 19363220 10.1182/blood-2009-02-204321 PMC2710920

[150] Liu, L. et al. Synergistic killing effects of PD-L1-CAR T cells and colorectal cancer stem cell-dendritic cell vaccine-sensitized T cells in ALDH1-positive colorectal cancer stem cells. *J. Cancer* **12**, 6629–6639 (2021). 34659553 10.7150/jca.62123 PMC8517999

[151] Rodriguez-Vida, A. et al. Safety and efficacy of avelumab plus carboplatin in patients with metastatic castration-resistant prostate cancer in an open-label Phase Ib study. *Br. J. Cancer* **128**, 21–29 (2023). 36289372 10.1038/s41416-022-01991-4 PMC9814154

[152] Tan, A. C. et al. Precursor frequency and competition dictate the HLA-A2-restricted CD8+ T cell responses to influenza A infection and vaccination in HLA-A2.1 transgenic mice. *J. Immunol.* **187**, 1895–1902 (2011). 21765016 10.4049/jimmunol.1100664

[153] Durántez, M. et al. Induction of multiepitopic and long-lasting immune responses against tumour antigens by immunization with peptides, DNA and recombinant adenoviruses expressing minigenes. *Scand. J. Immunol.* **69**, 80–89 (2009). 19144076 10.1111/j.1365-3083.2008.02202.x

[154] Doonan, B. P. et al. Peptide Modification Diminishes HLA Class II-restricted CD4(+) T Cell Recognition of Prostate Cancer Cells. *Int. J. Mol. Sci.* **23**, 15234 (2022). 36499557 10.3390/ijms232315234 PMC9738740

[155] Shi, Y. et al. Optimized mobilization of MHC class I- and II- restricted immunity by dendritic cell vaccine potentiates cancer therapy. *Theranostics* **12**, 3488–3502 (2022). 35547749 10.7150/thno.71760 PMC9065178

[156] Li, A. et al. A high-affinity T-helper epitope enhances peptide-pulsed dendritic cell-based vaccine. *DNA Cell Biol.* **30**, 883–892 (2011). 21612399 10.1089/dna.2011.1222

[157] Syyam, A. et al. Adenovirus vector system: construction, history and therapeutic applications. *Biotechniques* **73**, 297–305 (2022). 36475496 10.2144/btn-2022-0051

[158] Guo, Z. S. et al. Vaccinia virus-mediated cancer immunotherapy: cancer vaccines and oncolytics. *J. Immunother. Cancer* **7**, 6 (2019). 30626434 10.1186/s40425-018-0495-7 PMC6325819

[159] Soliman, H. et al. Oncolytic T-VEC virotherapy plus neoadjuvant chemotherapy in nonmetastatic triple-negative breast cancer: a phase 2 trial. *Nat. Med.* **29**, 450–457 (2023). 36759673 10.1038/s41591-023-02309-4

[160] Heery, C. R. et al. Docetaxel Alone or in Combination With a Therapeutic Cancer Vaccine (PANVAC) in Patients With Metastatic Breast Cancer: A Randomized Clinical Trial. *JAMA Oncol.* **1**, 1087–1095 (2015). 26291768 10.1001/jamaoncol.2015.2736 PMC6622177

[161] Ramlau, R. et al. A phase II study of Tg4010 (Mva-Muc1-Il2) in association with chemotherapy in patients with stage III/IV Non-small cell lung cancer. *J. Thorac. Oncol.* **3**, 735–744 (2008). 18594319 10.1097/JTO.0b013e31817c6b4f

[162] Dobosz, P. et al. The Intriguing History of Cancer Immunotherapy. *Front. Immunol.* **10**, 2965 (2019). 31921205 10.3389/fimmu.2019.02965 PMC6928196

[163] Derré, L. et al. Intravesical Bacillus Calmette Guerin Combined with a Cancer Vaccine Increases Local T-Cell Responses in Non-muscle-Invasive Bladder Cancer Patients. *Clin. Cancer Res.* **23**, 717–725 (2017). 27521445 10.1158/1078-0432.CCR-16-1189

[164] Zheng, J. H. et al. Two-step enhanced cancer immunotherapy with engineered Salmonella typhimurium secreting heterologous flagellin. *Sci. Transl. Med.* **9**, eaak9537 (2017). 28179508 10.1126/scitranslmed.aak9537

[165] Zuo, B. et al. Universal immunotherapeutic strategy for hepatocellular carcinoma with exosome vaccines that engage adaptive and innate immune responses. *J. Hematol. Oncol.* **15**, 46 (2022). 35488312 10.1186/s13045-022-01266-8 PMC9052531

[166] Cheng, K. et al. Bioengineered bacteria-derived outer membrane vesicles as a versatile antigen display platform for tumor vaccination via Plug-and-Display technology. *Nat. Commun.* **12**, 2041 (2021). 33824314 10.1038/s41467-021-22308-8 PMC8024398

[167] Yu, X. et al. Melittin-lipid nanoparticles target to lymph nodes and elicit a systemic anti-tumor immune response. *Nat. Commun.* **11**, 1110 (2020). 32111828 10.1038/s41467-020-14906-9 PMC7048802

[168] Chu, Y. et al. Lymph node-targeted neoantigen nanovaccines potentiate anti-tumor immune responses of post-surgical melanoma. *J. Nanobiotechnol.* **20**, 190 (2022). 10.1186/s12951-022-01397-7 PMC9006542 35418151

[169] Zhang, J. et al. Direct Presentation of Tumor-Associated Antigens to Induce Adaptive Immunity by Personalized Dendritic Cell-Mimicking Nanovaccines. *Adv. Mater.* **34**, e2205950 (2022). 36217832 10.1002/adma.202205950

[170] Yang, R. et al. A core-shell structured COVID-19 mRNA vaccine with favorable biodistribution pattern and promising immunity. *Signal Transduct. Target Ther.* **6**, 213 (2021). 34059617 10.1038/s41392-021-00634-z PMC8165147

[171] Persano, S. et al. Lipopolyplex potentiates anti-tumor immunity of mRNA-based vaccination. *Biomaterials* **125**, 81–89 (2017). 28231510 10.1016/j.biomaterials.2017.02.019 PMC5378555

[172] Welters, M. J. et al. Vaccination during myeloid cell depletion by cancer chemotherapy fosters robust T cell responses. *Sci. Transl. Med.* **8**, 334ra352 (2016). 10.1126/scitranslmed.aad8307 27075626

[173] Melief, C. J. M. et al. Strong vaccine responses during chemotherapy are associated with prolonged cancer survival. *Sci. Transl. Med.* **12**, eaaz8235 (2020). 32188726 10.1126/scitranslmed.aaz8235

[174] Bagchi, S. et al. Immune Checkpoint Inhibitors for the Treatment of Cancer: Clinical Impact and Mechanisms of Response and Resistance. *Annu. Rev. Pathol.* **16**, 223–249 (2021). 33197221 10.1146/annurev-pathol-042020-042741

[175] Awad, M. M. et al. Personalized neoantigen vaccine NEO-PV-01 with chemotherapy and anti-PD-1 as first-line treatment for non-squamous non-small cell lung cancer. *Cancer Cell* **40**, 1010–1026.e1011 (2022). 36027916 10.1016/j.ccell.2022.08.003

[176] Balar, A. V. et al. Atezolizumab as first-line treatment in cisplatin-ineligible patients with locally advanced and metastatic urothelial carcinoma: a single-arm, multicentre, phase 2 trial. *Lancet* **389**, 67–76 (2017). 27939400 10.1016/S0140-6736(16)32455-2 PMC5568632

[177] Teixeira, L. et al. A First-in-Human Phase I Study of INVAC-1, an Optimized Human Telomerase DNA Vaccine in Patients with Advanced Solid Tumors. *Clin. Cancer Res.* **26**, 588–597 (2020). 31558479 10.1158/1078-0432.CCR-19-1614

[178] Zanetti, M. et al. T cell memory and protective immunity by vaccination: is more better? *Trends Immunol.* **27**, 511–517 (2006). 16997631 10.1016/j.it.2006.09.004

[179] Billeskov, R. et al. The effect of antigen dose on T cell-targeting vaccine outcome. *Hum. Vaccine Immunother.* **15**, 407–411 (2019). 10.1080/21645515.2018.1527496 PMC6422501 30277831

[180] Wei, J. et al. The paradigm shift in treatment from Covid-19 to oncology with mRNA vaccines. *Cancer Treat. Rev.* **107**, 102405 (2022). 35576777 10.1016/j.ctrv.2022.102405 PMC9068246

[181] Duinkerken, S. et al. Glyco-Dendrimers as Intradermal Anti-Tumor Vaccine Targeting Multiple Skin DC Subsets. *Theranostics* **9**, 5797–5809 (2019). 31534520 10.7150/thno.35059 PMC6735376

[182] Chen, X. Emerging adjuvants for intradermal vaccination. *Int. J. Pharm.* **632**, 122559 (2023). 36586639 10.1016/j.ijpharm.2022.122559 PMC9794530

[183] Sun, Z. et al. Evaluation of Therapeutic Equivalence for the Follow-On Version of Intravenously Administered Non-Biological Complex Drugs. *Clin. Pharmacokinet.* **59**, 995–1004 (2020). 32328977 10.1007/s40262-020-00889-9

[184] Witzigmann, D. et al. Lipid nanoparticle technology for therapeutic gene regulation in the liver. *Adv. Drug Deliv. Rev.* **159**, 344–363 (2020). 32622021 10.1016/j.addr.2020.06.026 PMC7329694

[185] Stertman, L. et al. Starch microparticles as an adjuvant in immunisation: effect of route of administration on the immune response in mice. *Vaccine* **22**, 2863–2872 (2004). 15246622 10.1016/j.vaccine.2003.12.019

[186] Zhao, X. et al. Different protective efficacies of a novel antigen-specific DNA vaccine encoding chicken type II collagen via intramuscular, subcutaneous, and intravenous vaccination against experimental rheumatoid arthritis. *Biomed. Pharmacother.* **144**, 112294 (2021). 34653764 10.1016/j.biopha.2021.112294

[187] Agliardi, G. et al. Intratumoral IL-12 delivery empowers CAR-T cell immunotherapy in a pre-clinical model of glioblastoma. *Nat. Commun.* **12**, 444 (2021). 33469002 10.1038/s41467-020-20599-x PMC7815781

[188] Liu, J. Q. et al. Intratumoral delivery of IL-12 and IL-27 mRNA using lipid nanoparticles for cancer immunotherapy. *J. Control. Release* **345**, 306–313 (2022). 35301053 10.1016/j.jconrel.2022.03.021 PMC9133152

[189] Patel, R. B. et al. Development of an In Situ Cancer Vaccine via Combinational Radiation and Bacterial-Membrane-Coated Nanoparticles. *Adv. Mater.* **31**, e1902626 (2019). 31523868 10.1002/adma.201902626 PMC6810793

[190] Warrell, M. J. et al. An economical regimen of human diploid cell strain anti-rabies vaccine for post-exposure prophylaxis. *Lancet* **2**, 301–304 (1983). 6135830 10.1016/S0140-6736(83)90288-X

[191] Mould, R. C. et al. Enhancing Immune Responses to Cancer Vaccines Using Multi-Site Injections. *Sci. Rep.* **7**, 8322 (2017). 28814733 10.1038/s41598-017-08665-9 PMC5559552

[192] Johansen, P. et al. Antigen kinetics determines immune reactivity. *Proc. Natl Acad. Sci.* **105**, 5189–5194 (2008). 18362362 10.1073/pnas.0706296105 PMC2278203

[193] Walsh, N. C. et al. Humanized Mouse Models of Clinical Disease. *Annu. Rev. Pathol.* **12**, 187–215 (2017). 27959627 10.1146/annurev-pathol-052016-100332 PMC5280554

[194] Chuprin, J. et al. Humanized mouse models for immuno-oncology research. *Nat. Rev. Clin. Oncol.* **20**, 192–206 (2023). 36635480 10.1038/s41571-022-00721-2 PMC10593256

[195] De La Rochere, P. et al. Humanized Mice for the Study of Immuno-Oncology. *Trends Immunol.* **39**, 748–763 (2018). 30077656 10.1016/j.it.2018.07.001

[196] Chang, D. K. et al. Human anti-CAIX antibodies mediate immune cell inhibition of renal cell carcinoma in vitro and in a humanized mouse model in vivo. *Mol. Cancer* **14**, 119 (2015). 26062742 10.1186/s12943-015-0384-3 PMC4464115

[197] King, M. A. et al. Human peripheral blood leucocyte non-obese diabetic-severe combined immunodeficiency interleukin-2 receptor gamma chain gene mouse model of xenogeneic graft-versus-host-like disease and the role of host major histocompatibility complex. *Clin. Exp. Immunol.* **157**, 104–118 (2009). 19659776 10.1111/j.1365-2249.2009.03933.x PMC2710598

[198] Shultz, L. D. et al. Humanized mice in translational biomedical research. *Nat. Rev. Immunol.* **7**, 118–130 (2007). 17259968 10.1038/nri2017

[199] Danner, R. et al. Expression of HLA class II molecules in humanized NOD.Rag1KO.IL2RgcKO mice is critical for development and function of human T and B cells. *PLoS One* **6**, e19826 (2011). 21611197 10.1371/journal.pone.0019826 PMC3096643

[200] Najima, Y. et al. Induction of WT1-specific human CD8+ T cells from human HSCs in HLA class I Tg NOD/SCID/IL2rgKO mice. *Blood* **127**, 722–734 (2016). 26702062 10.1182/blood-2014-10-604777 PMC4751022

[201] Chen, K. S. et al. Bifunctional cancer cell-based vaccine concomitantly drives direct tumor killing and antitumor immunity. *Sci. Transl. Med.* **15**, eabo4778 (2023). 36599004 10.1126/scitranslmed.abo4778 PMC10068810

[202] Bonaventura, P. et al. Identification of shared tumor epitopes from endogenous retroviruses inducing high-avidity cytotoxic T cells for cancer immunotherapy. *Sci. Adv.* **8**, eabj3671 (2022). 35080970 10.1126/sciadv.abj3671 PMC8791462

[203] He, J. et al. Defined tumor antigen-specific T cells potentiate personalized TCR-T cell therapy and prediction of immunotherapy response. *Cell Res.* **32**, 530–542 (2022). 35165422 10.1038/s41422-022-00627-9 PMC9160085

[204] Zhang, W. et al. Patient-derived xenografts or organoids in the discovery of traditional and self-assembled drug for tumor immunotherapy. *Front. Oncol.* **13**, 1122322 (2023). 37081982 10.3389/fonc.2023.1122322 PMC10110942

[205] Hegde, P. S. et al. Top 10 Challenges in Cancer Immunotherapy. *Immunity* **52**, 17–35 (2020). 31940268 10.1016/j.immuni.2019.12.011

[206] Hanahan, D. et al. Accessories to the crime: functions of cells recruited to the tumor microenvironment. *Cancer Cell* **21**, 309–322 (2012). 22439926 10.1016/j.ccr.2012.02.022

[207] Pitt, J. M. et al. Targeting the tumor microenvironment: removing obstruction to anticancer immune responses and immunotherapy. *Ann. Oncol.* **27**, 1482–1492 (2016). 27069014 10.1093/annonc/mdw168

[208] Lutsiak, M. E. et al. Inhibition of CD4(+)25+ T regulatory cell function implicated in enhanced immune response by low-dose cyclophosphamide. *Blood* **105**, 2862–2868 (2005). 15591121 10.1182/blood-2004-06-2410

[209] Viaud, S. et al. Cyclophosphamide induces differentiation of Th17 cells in cancer patients. *Cancer Res.* **71**, 661–665 (2011). 21148486 10.1158/0008-5472.CAN-10-1259

[210] Mu, X. et al. Tumor-derived lactate induces M2 macrophage polarization via the activation of the ERK/STAT3 signaling pathway in breast cancer. *Cell Cycle* **17**, 428–438 (2018). 29468929 10.1080/15384101.2018.1444305 PMC5927648

[211] Zhao, S. J. et al. Macrophage MSR1 promotes BMSC osteogenic differentiation and M2-like polarization by activating PI3K/AKT/GSK3β/β-catenin pathway. *Theranostics* **10**, 17–35 (2020). 31903103 10.7150/thno.36930 PMC6929615

[212] Kloss, C. C. et al. Dominant-Negative TGF-β Receptor Enhances PSMA-Targeted Human CAR T Cell Proliferation And Augments Prostate Cancer Eradication. *Mol. Ther.* **26**, 1855–1866 (2018). 29807781 10.1016/j.ymthe.2018.05.003 PMC6037129

[213] Sharma, P. et al. The Next Decade of Immune Checkpoint Therapy. *Cancer Discov.* **11**, 838–857 (2021). 33811120 10.1158/2159-8290.CD-20-1680

[214] Eberhardt, C. S. et al. Functional HPV-specific PD-1(+) stem-like CD8 T cells in head and neck cancer. *Nature* **597**, 279–284 (2021). 34471285 10.1038/s41586-021-03862-z PMC10201342

[215] Lutz, E. A. et al. Intratumoral nanobody-IL-2 fusions that bind the tumor extracellular matrix suppress solid tumor growth in mice. *PNAS Nexus* **1**, pgac244 (2022). 36712341 10.1093/pnasnexus/pgac244 PMC9802395

[216] Nakao, S. et al. Intratumoral expression of IL-7 and IL-12 using an oncolytic virus increases systemic sensitivity to immune checkpoint blockade. *Sci. Transl. Med.* **12**, eaax7992 (2020). 31941828 10.1126/scitranslmed.aax7992

[217] Wang, G. et al. An engineered oncolytic virus expressing PD-L1 inhibitors activates tumor neoantigen-specific T cell responses. *Nat. Commun.* **11**, 1395 (2020). 32170083 10.1038/s41467-020-15229-5 PMC7070065

[218] Zhou, J. et al. VHL and DNA damage repair pathway alterations as potential clinical biomarkers for first-line TKIs in metastatic clear cell renal cell carcinomas. *Cell. Oncol. (Dordr.)* **45**, 677–687 (2022). 35834099 10.1007/s13402-022-00691-8 PMC9424144

[219] Yang, W. et al. Potentiating the antitumour response of CD8(+) T cells by modulating cholesterol metabolism. *Nature* **531**, 651–655 (2016). 26982734 10.1038/nature17412 PMC4851431

[220] Tse, S. W. et al. mRNA-encoded, constitutively active STING(V155M) is a potent genetic adjuvant of antigen-specific CD8(+) T cell response. *Mol. Ther.* **29**, 2227–2238 (2021). 33677092 10.1016/j.ymthe.2021.03.002 PMC8261085

[221] Hu, Z. et al. Personal neoantigen vaccines induce persistent memory T cell responses and epitope spreading in patients with melanoma. *Nat. Med.* **27**, 515–525 (2021). 33479501 10.1038/s41591-020-01206-4 PMC8273876

[222] Westcott, P. M. K. et al. Low neoantigen expression and poor T-cell priming underlie early immune escape in colorectal cancer. *Nat. Cancer* **2**, 1071–1085 (2021). 34738089 10.1038/s43018-021-00247-z PMC8562866

[223] Cheng, D. T. et al. Memorial Sloan Kettering-Integrated Mutation Profiling of Actionable Cancer Targets (MSK-IMPACT): A Hybridization Capture-Based Next-Generation Sequencing Clinical Assay for Solid Tumor Molecular Oncology. *J. Mol. Diagn.* **17**, 251–264 (2015). 25801821 10.1016/j.jmoldx.2014.12.006 PMC5808190

[224] Ott, P. et al. A Phase Ib Trial of Personalized Neoantigen Therapy Plus Anti-PD-1 in Patients with Advanced Melanoma, Non-small Cell Lung. *Cancer, or Bladder. Cancer* **183**, 347–362 (2020). 33064988 10.1016/j.cell.2020.08.053

[225] Shing, J. Z. et al. Precancerous cervical lesions caused by non-vaccine-preventable HPV types after vaccination with the bivalent AS04-adjuvanted HPV vaccine: an analysis of the long-term follow-up study from the randomised Costa Rica HPV Vaccine Trial. *Lancet Oncol.* **23**, 940–949 (2022). 35709811 10.1016/S1470-2045(22)00291-1 PMC9255557

[226] Boorjian, S. A. et al. Intravesical nadofaragene firadenovec gene therapy for BCG-unresponsive non-muscle-invasive bladder cancer: a single-arm, open-label, repeat-dose clinical trial. *Lancet Oncol.* **22**, 107–117 (2021). 33253641 10.1016/S1470-2045(20)30540-4 PMC7988888

[227] Engelhard, V. H. et al. MHC-restricted phosphopeptide antigens: preclinical validation and first-in-humans clinical trial in participants with high-risk melanoma. *J Immunother Cancer* **8**, e000262 (2020). 32385144 10.1136/jitc-2019-000262 PMC7228659

[228] Cai, Z. et al. Personalized neoantigen vaccine prevents postoperative recurrence in hepatocellular carcinoma patients with vascular invasion. *Mol. Cancer* **20**, 164 (2021). 34903219 10.1186/s12943-021-01467-8 PMC8667400

[229] Pai, J. A. et al. Lineage tracing reveals clonal progenitors and long-term persistence of tumor-specific T cells during immune checkpoint blockade. *Cancer Cell* **41**, 776–790.e777 (2023). 37001526 10.1016/j.ccell.2023.03.009 PMC10563767

[230] Danilova, L. et al. The Mutation-Associated Neoantigen Functional Expansion of Specific T Cells (MANAFEST) Assay: A Sensitive Platform for Monitoring Antitumor Immunity. *Cancer Immunol. Res.* **6**, 888–899 (2018). 29895573 10.1158/2326-6066.CIR-18-0129 PMC6072595

[231] Harrison, R. P. et al. Decentralised manufacturing of cell and gene therapy products: Learning from other healthcare sectors. *Biotechnol. Adv.* **36**, 345–357 (2018). 29278756 10.1016/j.biotechadv.2017.12.013

[232] Palmer, C. D. et al. Individualized, heterologous chimpanzee adenovirus and self-amplifying mRNA neoantigen vaccine for advanced metastatic solid tumors: phase 1 trial interim results. *Nat. Med.* **28**, 1619–1629 (2022). 35970920 10.1038/s41591-022-01937-6

[233] De Keersmaecker, B. et al. TriMix and tumor antigen mRNA electroporated dendritic cell vaccination plus ipilimumab: link between T-cell activation and clinical responses in advanced melanoma. *J Immunother Cancer* **8**, e000329 (2020). 32114500 10.1136/jitc-2019-000329 PMC7057443

[234] Aggarwal, C. et al. Immunotherapy Targeting HPV16/18 Generates Potent Immune Responses in HPV-Associated Head and Neck Cancer. *Clin. Cancer Res.* **25**, 110–124 (2019). 30242022 10.1158/1078-0432.CCR-18-1763 PMC6320307

[235] Chen, Z. et al. A Neoantigen-Based Peptide Vaccine for Patients With Advanced Pancreatic Cancer Refractory to Standard Treatment. *Front. Immunol.* **12**, 691605 (2021). 34484187 10.3389/fimmu.2021.691605 PMC8414362

[236] Mueller, S. et al. Mass cytometry detects H3.3K27M-specific vaccine responses in diffuse midline glioma. *J. Clin. Invest.* **130**, 6325–6337 (2020). 32817593 10.1172/JCI140378 PMC7685729

[237] Platten, M. et al. A vaccine targeting mutant IDH1 in newly diagnosed glioma. *Nature* **592**, 463–468 (2021). 33762734 10.1038/s41586-021-03363-z PMC8046668

[238] Kloor, M. et al. A Frameshift Peptide Neoantigen-Based Vaccine for Mismatch Repair-Deficient Cancers: A Phase I/IIa Clinical Trial. *Clin. Cancer Res.* **26**, 4503–4510 (2020). 32540851 10.1158/1078-0432.CCR-19-3517

